# Mine or ours? Neural basis of the exploitation of common-pool resources

**DOI:** 10.1093/scan/nsac008

**Published:** 2022-02-01

**Authors:** Mario Martinez-Saito, Sandra Andraszewicz, Vasily Klucharev, Jörg Rieskamp

**Affiliations:** International Laboratory of Social Neurobiology, Institute of Cognitive Neuroscience, HSE University, Russian Federation, Moscow 101000, Russia; Department of Humanities, Social and Political Sciences, ETH Zurich, Zurich 8006, Swiss Confederation; Department of Psychology, University of Basel, Basel 4055, Swiss Confederation; International Laboratory of Social Neurobiology, Institute of Cognitive Neuroscience, HSE University, Russian Federation, Moscow 101000, Russia; Department of Psychology, University of Basel, Basel 4055, Swiss Confederation

**Keywords:** common goods, tragedy of the commons, social competition, ventral striatum, reinforcement learning, social comparison

## Abstract

Why do people often exhaust unregulated common (shared) natural resources but manage to preserve similar private resources? To answer this question, in this study we combine a neurobiological, economic and cognitive modeling approach. Using functional magnetic resonance imaging on 50 participants, we show that a sharp decrease of common and private resources is associated with deactivation of the ventral striatum, a brain region involved in the valuation of outcomes. Across individuals, when facing a common resource, ventral striatal activity is anticorrelated with resource preservation (less harvesting), whereas with private resources the opposite pattern is observed. This indicates that neural value signals distinctly modulate behavior in response to the depletion of common *vs* private resources. Computational modeling suggested that overharvesting of common resources was facilitated by the modulatory effect of social comparison on value signals. These results provide an explanation of people’s tendency to over-exploit unregulated common natural resources.

The sustainability of environmental resources is of worldwide concern in the twenty-first century. Currently, the world faces a rapid decline of many natural resources, such as fish stocks, clean air and primeval forests (Ostrom, 2009). The collapse of the Atlantic northwest cod fisheries in 1992 (Myers *et al.*, 1997) lead to the endangering of the Atlantic cod and the devastation of fishing communities in Newfoundland; its fisheries have not recovered to this day despite a moratorium on fishing. This is just one of the many instances of overexploitation of natural resources plaguing the environment (Rosser and Mainka, 2002). Thus, understanding how modern human cooperative behavior forms in shared resource systems such as fishing grounds (Klein *et al.*, 2017), water and timber in the context of social heterogeneity (Sugiarto *et al.*, 2017) is an issue of vital importance. In the present article, we explore the neurobiological underpinnings of shared resource overexploitation. We combine neurobiological, economic and computational approaches to explain why humans treat a resource differently in a competitive social environment as compared to a private environment.

Economic theory predicts the overexploitation of common resources by self-interested people. This claim is illustrated by the ‘tragedy of the commons’ (Hardin, 1968): a dilemma in which multiple individuals, acting independently and rationally, will ultimately deplete a shared, limited resource even if it is against their long-term interest. For example, a group of people sharing fishing grounds often realize that they greatly benefit from increasing their own catch. Yet if every person focuses too much on his or her own profit the fish stock becomes eventually depleted (Osten *et al.*, 2017). This social dilemma is commonly conceptualized as a *common-pool resource* (CPR) dilemma. In such a situation a natural or urban system generates benefits that can be consumed by individuals who cannot be excluded from consumption (Ostrom, 1990). According to economic theory, non-excludable goods that anyone can enter and/or harvest are likely to be overharvested and destroyed. However, behavioral economics also gives many examples in which people behave fairly and cooperatively contrary to the standard self-interest model (Fehr and Schmidt, 1999): under some conditions, in particular in two-person interactions, people often show high rates of cooperation (Fehr and Gachter, 2000). Why, then, is it so difficult even for cooperative people to overlook short-term benefits and sustain CPRs for larger, long-term benefits?

It has been shown that overharvesting is particularly prevalent in social groups containing a substantial number of ‘free riders’, that is, people who take benefits without paying any costs (Camerer, 2003). One explanation for the tendency to overharvest CPRs refers to people’s social preference for equity and reciprocal cooperation (Fehr and Schmidt, 1999; Falk and Fischbacher, 2006): If others are cooperative, then people act cooperatively, but if others free ride, people correspondingly retaliate. Accordingly, in a group that contains few free riders, the average consumption of the CPR will be higher than the consumption of its cooperative members. If cooperative members perceive that returns from the common resource are meagerer than expected (Brandt *et al.*, 2012), or even if they behave reciprocally by choosing the average consumption rate for the future, an upward spiral of consumption is set off and CPRs are overexploited (Fehr and Fischbacher, 2003). Thus, overexploitation can result even for cooperative people who monitor their own and their conspecifics’ behavior and act reciprocally.

Here we hypothesize that the brain dopaminergic system, a set of brain areas involved in reward and performance monitoring, not only continuously monitors our own outcomes (Osten *et al.*, 2017) during CPR interactions but also monitors the outcomes of others. The dopaminergic system has been previously implicated in *social comparison* (Fliessbach *et al.*, 2007; Dvash *et al.*, 2010; Bault *et al.*, 2011) —the spontaneous tendency to compare one’s own behavior with that of others (Festinger, 1954). We suggest that when dealing with CPRs, the dopaminergic system continually compares personal outcomes with the outcomes of others. In case of noticing free-riding behavior that results in inequality, the dopaminergic system might facilitate an overharvesting response. When dealing with private resources, however, the dopaminergic system would monitor deviations from outcomes that maintain long-term resource sustainability. More specifically, we hypothesize that individual overexploitation tendencies have to be depicted in the ventral striatum activity.

A recent meta-analysis has identified consistent involvement of the ventral striatum in social comparison (Luo *et al.*, 2018). Furthermore, neuroimaging studies suggest that the ventral striatum processes social rewards (Delgado, 2007; Izuma *et al.*, 2008, 2010; Meshi *et al.*, 2013), on top of its established crucial role in general reward processing. Importantly, it has been hypothesized that when people detect differences between self and others, social (norm) prediction errors might be detected in the ventral striatum (Klucharev *et al.*, 2009; Luo *et al.*, 2018; see also Montague and Lohrenz, 2007, for the concept of ‘norm prediction errors’). Thus, by analyzing neural activity in the striatum we can investigate why people adopt suboptimal patterns in the consumption of CPR because (1) its response to learning signals under social and nonsocial contexts will help to elucidate the way social comparisons affect the encoding of reward and (2) it provides a means to validate computational models of learning.

To find a computational explanation for the increasing CPR depletion, we developed a computational model that posits a reward prediction error (RPE) that compares a person’s own outcome with the harvesting behavior of conspecifics. We hypothesize that the ventral striatum is associated with this RPE signal. The suggested model of social comparison follows the classic idea of people’s social preference for equity (Fehr and Schmidt, 1999; Falk and Fischbacher, 2006), with the difference that we assume that receiving more than the competitors induces social preferences for equity (see e.g. Fliessbach *et al.*, 2007, for a similar concept). Thus, we hypothesize that social comparison is encoded in the neural learning signal that facilitates overharvesting of the common natural resources.

## Materials and methods

### Participants

After informed consent, 50 healthy, right-handed students participated in the neuroimaging experiment (aged 18–32 years, mean 23.4 years, 26 females). None of the participants reported a history of drug abuse, head trauma, neurological or psychiatric illness. Participants were randomly assigned to the social or private (nonsocial) condition (*N* = 24 for the social and *N* = 26 for the nonsocial condition). This sample size was chosen to yield an approximate statistical power of 80% (Murphy and Garavan, 2004; Friston, 2012) assuming an approximate Cohen’s *d* effect size of 0.7 for a conventional functional magnetic resonance imaging (fMRI) analysis, i.e. linear mixed-effects analysis using a 5% family-wise error rate (FWER) threshold from random field theory (Poldrack *et al.*, 2017). Three participants were rejected from the fMRI analysis due to head motion exceeding 3 mm; one participant was excluded due to misunderstanding the instructions and a high error rate (*N* = 22 for the social and *N* = 24 for the nonsocial condition). The study was approved by the local ethics committee of the Canton of Basel City, Switzerland.

### Task design

Participants had to manage CPR in the form of fish stock, by imagining that they were fishing by a lake together with two other fishermen. Their task was to collect as much fish as possible and each collected fish led to a monetary payoff (0.25 Swiss Francs per fish). In every trial, participants decided between three possible net sizes for fishing: one, two or three. Depletion of the resource (fishing out the lake) was caused by their own behavior and the behavior of two other anonymous players present in the room. Participants were informed that although the fish stock in the lake decreases by fishing, it is also replenished naturally; the maximum capacity of the lake was 16 fish. At the end of every trial, the number of fish was multiplied by 1.5 (rounded down) and shown to the participant. This fish stock was carried over for the next trial. If the lake was fished out, the session ended automatically. The instructions clearly explained that the number of fish taken out could increase, sustain or decrease the fish population, and that if the total number of fish collected by the three participants was less than six, the fish population would increase over the trials; otherwise, the fish population would decrease over the trials. If the total number of fish collected by the three people was six, the fish population would stay constant. A net size of two fish corresponded to a cooperative/sustainable level of harvesting, whereas three represented overharvesting and one led to replenishment. Participants were told that their pre-recorded opponents played under the same conditions; in particular, their opponents were motivated by the same payment stipulation. The opponents were pre-recorded surrogates from a pilot behavioral study [Supplementary Information (S.I.), section A]. In the nonsocial condition, the task structure was kept identical, but the instructions replace the opponents by natural action, i.e. fish stock decrease was attributed to ‘migration’ (S.I., section E). The participants of the pilot behavioral study were not informed about the follow-up fMRI study, but they gave permission to use their (anonymized) data in later studies. The experiment started with a short training session. On average, participants earned 33.3 Swiss Francs (30 SFR as participation fee and 3.3 SFR as monetary payoff). Under the game-theoretic assumption that participants play rationally (with the sole goal to maximize utility), it can be shown that chosen net sizes should be always maximal in the social condition, and rapidly increase from one to the maximum net size by the middle of the session the in the nonsocial condition. However, players’ behavior strongly deviated from it. This is analyzed in sections A, B and C of S.I.

**Fig. 1.**
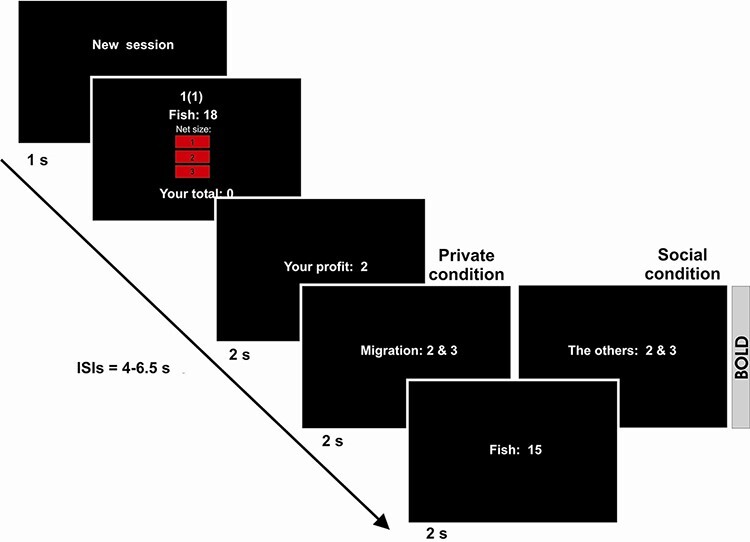
The nonsocial and social versions of the CPR task. The sequence of events within a trial is shown. Participants removed 1, 2 or 3 fish from the CPR and observed either ‘migration’ of the fish into neighboring lakes (nonsocial condition) or ‘fishing’ by two pre-recorded participants (social condition). At the end of each trial and at the beginning of the next trial participants were informed about the remaining number of fish in the CPR. ISI: inter-stimulus interval.

To avoid any demand effects and suspicion toward the two different (but structurally identical) conditions, we implemented a between-subject design: participants were randomly assigned to either a social or a nonsocial condition. Importantly, this was done because in a within-subject design the risk of the participant realizing that they go through exactly the same scenario, but received a different ‘story’ would be too high. Previous studies (e.g. Levin *et al.*, 1987) indicate that the initial framing setup carries over despite subsequent frame changes. Participants played 16 sessions in total (maximum 8 trials per session). In every trial, participants decided between three possible net sizes for fishing with one, two or three fish, respectively (Figure 1). Their task was to collect as many fish as possible, and each collected fish led to a monetary payoff (0.25 Swiss francs per fish). In the social version of the experiment (social condition), two other participants pre-recorded in a behavioral study (see S.I., section B) also decided between the three net sizes. In the nonsocial version of the experiment (nonsocial condition), the same number of fish ‘migrated’ to two neighboring lakes. Importantly, the change of the resources due to the two other pre-recorded participants or the ‘migration’ to the two neighboring lakes was identical in both conditions. This replicated the results of the behavioral study (S.I., section B). Although pre-recorded data are often used in fMRI studies (e.g. Engelmann *et al.*, 2019), it is important to assess their effect on the results: in a pilot study, the behavior of players performing a simultaneous and interactive version of the same task (S.I., section A) was similar to the results of the fMRI study. To preserve the interactive nature of the game, participants were told that their choices had real, but delayed, consequences for their counterparts, who were sent additional payments according to their decisions made in the scanner after completion of the experiment. We verified that participants had understood this during debriefing. Results from an exit questionnaire assessing participants’ perception of the social nature of their interactions during the main experiment indicated that participants trusted our experimental instructions and believed that they were interacting with a real partner (for the same approach, see Engelmann *et al.*, 2019).

### fMRI data acquisition

Functional MRI was performed with ascending slice acquisition using a T2*-weighted echo-planar imaging sequence (3 T Siemens Magnetom Verio whole-body MR unit equipped with a twelve-channel head coil; 40 axial slices; volume repetition time (TR), 2.28 s; echo time (TE), 30 ms; 80° flip angle; slice thickness 3.0 mm; field of view 228 mm; slice matrix 76 × 76). For structural MRI, we acquired a T1- weighted MP-RAGE sequence (176 sagittal slices; volume TR 2.0 s; TE, 3.37 ms; 8° flip angle; slice matrix 256 × 256; slice thickness, 1.0 mm; no gap; field of view, 256 mm).

### fMRI data analysis

Image analysis was performed with SPM12 (Friston *et al.*, 1994). The first four EPI volumes were discarded to allow for T1 equilibration, and the remaining images were realigned to the first volume. Images were then corrected for differences in slice acquisition time, spatially normalized to the Montreal Neurological Institute (MNI) T1 template, resampled to 3 × 3 × 3 mm^3^ voxels, and spatially smoothed with a Gaussian kernel of 8 mm full-width at half-maximum. Data were high-pass filtered (cutoff at 1/128 Hz). All five-time windows (frames) of the trial were modeled separately in the context of the general linear model implemented in SPM. The last trial in each session was excluded from the analysis because participants had no incentive to preserve the resource. Motion parameters were included in the GLM as covariates of no interest.

We constructed separate regressors for different scenarios of resource decrease due to either fishing by others or migration: feedback upon a sharp (6 fish) loss, conducive to resource depletion, or a moderate (2, 3 or 4 fish) loss, which is always sustainable, were modeled as individual hemodynamic responses (2 s after trial onset). Based on the ensuing parameter estimates, contrasts of interest were generated. The contrast images were then entered into a second-level analysis with the participant as a random grouping factor. Here, the individual GLM regressor coefficients implied by the first level contrasts are used to draw inferences about effects at the level of the population from which individual coefficients were sampled. Specifically, we used a conventional summary statistics approach (Holmes and Friston, 1998), which assumes homogeneous within-subject variance. To examine regions monitoring perceived CPR fluctuations in a separate analysis, one regressor specified at feedback events, regardless of specific scenarios of resource depletion, was parametrically modulated by the fish stock remaining in the lake each trial during the trial end screen showing the fish stock size. Besides these regressors of interest, to control for potential confounds, the GLM regressor matrix included the following events: choice stage, outcome stage (boxcar and its parametric modulation by the number of fish) and the stock size screen ending each trial (cf. Figure 1). In addition, different cognitive models were used to analyze the data: to examine regions associated with RPE, the regressor associated with the feedback event was parametrically modulated by the RPE that was calculated for each trial based on a social or nonsocial version of the reinforcement learning models (see below for details).

We focused on the ventral striatum and the ventromedial prefrontal cortex (vmPFC) because they belong to the brain’s valuation system through their essential role in valuation and reward-based learning (Levy and Glimcher, 2012; Bartra *et al.*, 2013). Similarly to (Bartra *et al.*, 2013), we built vmPFC and striatum regions of interest (ROI) with labels from the 1 mm anatomic atlas parcellation resolution of (Rolls *et al.*, 2015) by taking the bilateral union of gyrus rectus, medial orbitofrontal, anterior orbitofrontal and posterior orbitofrontal regions for vmPFC. The volume of the bilateral vmPFC ROI was 39.896 cm^3^. We created a bilateral ROI in the ventral striatum with a 10 mm-radius sphere with center in MNI coordinates [*x* = ±12, *y *= 11, *z* = −6], corresponding to the peak meta-analytic statistic location for a positive effect of subjective value on BOLD signal, along with its contralateral hemisphere homologue, based on an fMRI meta-analysis of subjective value neural correlates (Bartra *et al.*, 2013). To control for Type I errors in whole-brain analyses we set the cluster-forming threshold at *P* < .001. ROI statistics were calculated using the Matlab toolbox Marsbar v0.44 (Brett *et al.*, 2002). Brain images were created with MRIcroGL v1.2 (Rorden and Brett, 2000) and SPM (Friston *et al.*, 1994).

### Social learning model

To explain the effect of the social context on fishing behavior in the CPR task, we used a variation of the reinforcement learning model (Sutton and Barto, 1998). The model assigns to each choice option a subjective expectation value, which is updated on a trial-by-trial basis. The probability *p_t_(i)* of choosing an option (net size) *i* at time *t* depends on the option’s subjective expectations, as specified by a softmax choice rule:

(1) $${p_{\rm{t}}}\left( i \right) = {{exp\left[ {{\beta} \cdot {Q_{t - 1}}\left( i \right)} \right]} \over {\sum {exp}\left[ {{\beta} \cdot {Q_{t - 1}}\left(\,j \right)} \right]}}$$

where *Q_t-1_(i)* is the current subjective (expected) value for choice *i* and β > 0 is the inverse t emperature parameter that determines the choice sensitivity of the chosen option with the highest subjective value. Large values of β signify that the option with the highest subjective value is chosen with a high probability, whereas low values of *β* signify high choice randomness. The subjective values *Q_t_(i)* were updated each trial after the participant made a decision and obtained feedback about the two competitors’ decisions (social condition) or migration (nonsocial condition). Thus, in all trials *t—*with *t* an integer between 1 and 8, included*—*we calculated the subjective value for each choice *i*:

(2) $${Q_t}\left( i \right) = {Q_{t - 1}}\left( i \right){\rm{ + \alpha }}\left( {{R_{{\rm{i,t}}}} - {Q_{t - 1}}\left( i \right)} \right)$$

where *R*_*i, t*_ is the participant’s reinforcement from the current choice and where (*R_i, t_—Q_t-1_*(i)) represents the RPE between the participant’s expectation and the actual reward. The reinforcement *R_t_(i)* in a given trial *t* is a weighted sum between the direct reward from the resource-derived reward and a social comparison component *SocComp_i, t_*:

(3) $${R_{{\rm{i,t}}}} = \left( {1 - {\theta_s}} \right) \cdot OwnPayof\ {f_{{\rm{i,t}}}} + {\theta_s} \cdot SocCom{p_{i,t}}$$

where 0 ≤* θ_s_* *≤ *1 indicates the relative weight given to the personal payoff and the social comparison value. The social comparison component *SocComp_i, t_* was calculated at every trial *t* for the three net sizes *i* as the difference between the participant’s own payoff $OwnPayof\ {f_{i,t}}$ and the average payoff of the other players $\langle{OthersPayof\ {f_t}}\rangle$:

(4) $$SocCom{p_{i,t}} = OwnPayof\ {f_{{\rm{i,t}}}} - \langle{OthersPayof\ {f_t}}\rangle$$

Equation (4) weighs the social and nonsocial components of reward with a free parameter *θ_s_* while defining social reward as the excess reward with respect to other players, which incorporates the postulate that people try to minimize disadvantageous inequity of outcomes (Fehr and Schmidt, 1999).

The parameter a∈[0,1] denotes the learning rate. Unlike standard reinforcement models (Sutton and Barto, 1998), we assumed not only that the expectation of the chosen option was updated, but also that the expectations of the two unchosen options were updated (Camerer and Ho, 1999; Coricelli *et al.*, 2005). Therefore, our model represents a variant of a standard reinforcement model with the difference of updating all options as suggested by fictive updating (Montague *et al.*, 2006) and recent work in the neuroimaging literature on fictitious prediction errors (Hampton *et al.*, 2007, 2008; Glascher *et al.*, 2009). For the non-chosen option, a counterfactual payoff (given by a hypothetical choice) was used to determine the fictive prediction error. For the fMRI analysis, we nevertheless used the prediction error of the chosen option as a parametric modulator.

Participants believed that each session they played in a new environment with different opponents or migration dynamics. To estimate the a priori expectations about the outcome of choices *Q_0_(i)* when a participant starts fishing in the first trial (*t *= 1), we calculated the actual frequencies of choosing each net size in the first trials of all sessions multiplied by four:

(5) $${Q_0}\left( i \right) = 4 \cdot {\left( {{{{n_i}} \over N}} \right)_{t = 1}}$$

where (*n_i/_*N)_t=1_ is the number of particular choices (e.g. net size in the first trial in each session) divided by the total number of sessions. The expected frequencies were multiplied by four to scale the initial expectations to the range of rewards that could be obtained in the task (number of fish: 1, 2 and 3). Thus, ${Q_0}\left( i \right)$ is a constant in the model. We further hypothesized that in the social condition people not only take their personal payoff into account but also compare their payoff with the other players’ payoffs to determine an overall reinforcement (following social preference models, e.g. Fehr and Schmidt, 1999). Therefore, the reinforcement of an outcome results from the personal payoff and a social comparison component. According to the social comparison component of our model, the participant received a negative reinforcement if the participant’s payoff was lower than the other players’ average payoff. When the participant took more than the other players took on average this led to a reward. The *social learning model* has three free parameters: the *learning rate* α, the *inverse temperature* β and the *social comparison weight* θ_s_. We designate the RPE derived from the social learning model as the social RPE (sRPE).

### Sustainable nonsocial learning model

We suggest that in the nonsocial condition, people take their personal payoff into account but are also motivated to sustain the resource in the long term. Therefore, the reinforcement of an outcome would result from the weighted personal payoff and a sustainability component *SustComp_i, t_*:

(6) $${R_{i,t}} = \left( {1 - {\theta_n}} \right) \cdot OwnPayof\ {f_{i,t}} + {\theta_n} \cdot SustCom{p_{i,t}}$$

*SustComp_i, t_* is the negative absolute value of the difference between the optimal (sustainable) fish stock decrease (i.e. *SustainableCatch* = six fish) and the sum of the actual number of fish taken out (i.e. *OwnPayoff_i, t_*) and migrated to another lake (i.e. *Outflow_i, t_*):

(7) $$SustCom{p_{i,t}} = - \left| {SustainableCatch - OwnPayof\ {f_{{\rm{i,t}}}} - Outflo{w_{{\rm{i,t}}}}} \right|$$

This implies that the value of the sustainability component was either zero (when the sum of fish taken from the resource was equal to the *sustainable* number) or negative (when ‘too many’ *or* ‘too few’ were extracted). The rationale behind this ‘punishment’ was that taking ‘too few’ misses a chance to profit and taking ‘too many’ harms the sustainability of the resource and thereby jeopardizes future payoffs. Thus, according to the sustainability component, a participant was penalized for taking too many from the resource if the migration was large, and similarly, participants were also penalized for taking too few if the migration was small. Importantly, in the social and the nonsocial conditions, participants were clearly informed in the instructions of the experiment that when the resource decreased by six fish the number of fish in the lake would stay constant over time. The sustainable nonsocial learning model also had three free parameters: the learning rate α, the inverse temperature β and the sustainability weight θ_n_. We designate the RPE derived from this model as nonsocial RPE (nRPE).

### Rescorla–Wagner and Fehr–Schmidt models

We also tested the previous two learning models against another two competing models (Table 1): a vanilla reinforcement learning model (RW model, Rescorla and Wagner, 1972) and a modified inequity aversion model (FS; Fehr and Schmidt, 1999). The RW model only considers the personal payoffs in the task as reinforcement and had no sustainability component, so in essence, it is an asocial model, i.e. it is unconcerned with the sociality of rewards. Thus, the RW model is nested within the (social or nonsocial) learning model when setting the weight θ_s_ (or θ_n_) of the corresponding models equal to zero. The FS model was identical to the social learning model, with the exception that the comparison component was defined as:

(8) $${C_{i,t}} = \;OwnPayof{f_{i,t}} - {\delta ^ - }\sum\limits_{j = 1}^2 {{\rm{max}}} \left[ {OthersPayof\;{f_{i,j,t}} - OwnPayof\;{f_{i,t}},0} \right] - {\delta ^ + }\sum\limits_{j = 1}^2 {{\rm{max}}} \left[ {OwnPayof\;{f_{i,t}} - OthersPayof\;{f_{i,j,t}},0} \right]$$

where the δ’s were the advantageous (δ^+^) and disadvantageous inequality (δ^−^) coefficients (Fehr and Schmidt, 1999).

**Table 1.** Learning models (*N* = number of participants). The value updating equation ${Q_t}\left( i \right) = {Q_{t - 1}}\left( i \right){\rm{ + \alpha }}\left( {{R_{{\rm{i,t}}}} - {Q_{t - 1}}\left( i \right)} \right)$ and policy (action selection) equation${p_{\rm{t}}}\left( i \right) = {{exp\left[ {{\beta } \cdot {Q_{t - 1}}\left( i \right)} \right]} \over {\mathop \sum {exp}\left[ {{\beta} \cdot {Q_{t - 1}}\left( j \right)} \right]}}$ are common to all models (except the null)

Model type	Number of parameters	Reinforcement (reward) component
Null (baseline)	*N*	None
Social learning	3N	Payoff and Social comparison ${R_{{\rm{i,t}}}} = \left( {1 - {\theta_s}} \right) \cdot OwnPayof\ {f_{{\rm{i,t}}}} + {\theta_s} \cdot SocCom{p_{i,t}}$ where $SocCom{p_{i,t}} = OwnPayof\ {f_{{\rm{i,t}}}} - \langle{OthersPayof\ {f_t}}\rangle$
Sustainable nonsocial learning	3N	Payoff and Sustainability ${R_{i,t}} = \left( {1 - {\rm{\sigma }}} \right) \cdot OwnPayof\ {f_{i,t}} + {\rm{\sigma }} \cdot {S_{i,t}}$ where $SustCom{p_{i,t}} = - \left| {SustainableCatch - OwnPayof\ {f_{{\rm{i,t}}}} - Outflo{w_{{\rm{i,t}}}}} \right|$
Model free reinforcement learning (Rescorla–Wagner)	2N	Payoff ${R_{i,t}} = OwnPayof\ {f_{i,t}}$
Inequity aversion (Fehr–Schmidt)	5N	Payoff and Social comparison with inequity aversion ${R_{{\rm{i,t}}}} = \left( {1 - {\theta_s}} \right) \cdot OwnPayof\ {f_{{\rm{i,t}}}} + {\theta_s} \cdot SocCom{p_{i,t}}$ where ${SocCom{p_{i,t}} = OwnPayof\ {f_{i,t}}{\rm{ - }}{\delta ^{ + }}\mathop \sum \limits_{j = 1}^2 {\rm{max}}\left[ {OthersPayof \ {f_{i,j,t}} - OwnPayof\ {f_{i,t}},{\rm{ 0}}} \right]}$ $\qquad\qquad\qquad{{\rm{ - }}{\delta ^{\rm{ - }}}\mathop \sum \limits_{j = 1}^2 {\rm{max}}\left[ {ownPayof\ {f_{i,t}} - OthersPayof\ {f_{i,j,t}},{\rm{ 0}}} \right]}$

### Evaluation of the models

**Fig. 2.**
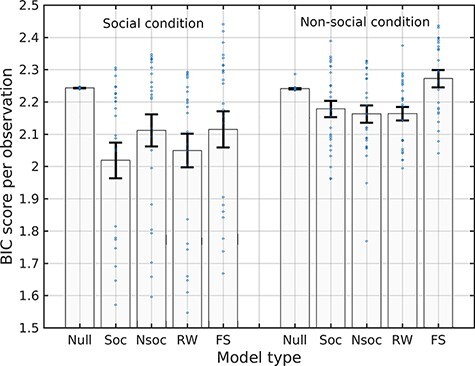
Distribution of goodness of fit, as measured by BIC scores across subjects per observation, by model type and experimental condition. Abscissa labels: Null model (Null) Social model (Soc), Nonsocial model (Nsoc), Rescorla–Wagner model (RW), Fehr–Schmidt inequity aversion model (FS). Bar heights correspond to BIC score group means and error bars indicate s.e.m.

Initially, we evaluated the models by comparing them to the null (baseline) model, which assumed a uniformly random choice of the three net sizes (i.e. predicting a uniform choice probability of 1/3) using the Bayesian Information Criterion (BIC; Schwarz, 1978). BIC scores are an approximation to model log-evidence that accounts for model complexity. The average BIC per observation was 2.075 (s.d. = 0.265) for the social learning model and 2.080 (s.d. = 0.235) for the sustainable nonsocial learning model as compared to the average BIC of 2.242 for the null (baseline) model. A mixed ANOVA with the participant as a grouping random effect and model type as a fixed effect factor showed that on average the learning models described the data better than the baseline model (Social-Null: df = 1, F = 19.78, *P = *4.98e-5; Nonsocial-Null: df = 1, F = 23.73, *P* = 1.2e-5). In the social condition, to examine individual differences, the social learning model was better than the null model for 75% of the participants according to BIC and in the nonsocial condition, the sustainable nonsocial learning model was better than the baseline for 69% of the participants. Thus, although both learning models on average did better than the baseline model, for some participants the better fit of the learning models in comparison to the baseline model was not large enough when taking model complexity into account. A mixed ANOVA with the participant as grouping random effect and model type as fixed effect confirmed that there was a difference between the learning models and the null model (Social-Null: df = 1, F = 19.78, *P = *4.98e-5; Nonsocial-Null: df = 1, F = 23.73, *P* = 1.2e-5). To further examine the empirical validity of the models, we compared the social learning model with the sustainable nonsocial learning model by ‘cross-fitting’ both models: we fit the nonsocial model to the participants in the social condition and the social learning model to the participants in the nonsocial condition. This approach should show that the models were unsuitable when applied to incongruent conditions (i.e. nonsocial model fit to the social condition). We further compared these models with the RW and FS models (Table 1); the social learning model performed better than the two competing models in the social condition, but the sustainable nonsocial learning model was matched by the RW model in the nonsocial condition (Figure 2). We also compared the BIC scores of the social *vs* FS model and nonsocial *vs* RW model, we added the individual BIC scores of each participant for each model and compared the sums (Table 2). Since BIC scores can be hard to interpret, we also calculated McFadden pseudo-R-squares (McFadden, 1974) and balanced accuracy (Brodersen *et al.*, 2010), where the (non)social model won in the (non)social condition. For the balanced accuracy computation, we assumed RW to be a nonsocial model and FS to be a social model. Again, the relevant models won in the relevant conditions in a Bayesian model selection analysis (Table 2) as measured by model frequency (expected multinomial parameters, i.e. the probability that each model generated the observed data) and exceedance probability (Stephan *et al.*, 2009; Daunizeau *et al.*, 2014). Finally, we also performed a model recovery analysis, to show that our approach to select the model is reliable in this context. The learning models were used to generate behavior, faced with the same pre-recorded surrogates as human participants. This requires small modifications in the algorithm that furnish it with a policy to select actions on the basis of learned Q-values (Eq. 1). This enacts artificial players that behave as specified by each of the four learning algorithms described in this section. Each of the learning models was run 50 times (matching the number of participants) pitted against the same pre-recorded dataset as human subjects; the produced simulated data were then fitted exactly as behavioral data were (see next subsection). The analysis results are shown in Table 5, and the resulting fitted parameters are can be found in the S.I. (Figure S3).

**Table 2.** Aggregate BIC scores, model frequency, exceedance probability, McFadden pseudo-R-squared and balanced accuracy for each learning model and condition

Measure	Condition	Null	Social	Nonsocial	Rescorla–Wagner	Fehr–Schmidt	t
BIC scores	Nonsocial	6456.6	6247.1	6208.1	6208.9	6512.8	–
	Social	5412.5	4895.0	5104.2	4957.0	5114.7	–
Model frequency	Nonsocial	0.1662	0.2159	0.2358	0.2333	0.1489	–
	Social	0.1268	0.2802	0.1804	0.2357	0.1769	–
Exceedance probability	Nonsocial	0.0884	0.2312	0.3170	0.3040	0.0594	–
	Social	0.0293	0.4952	0.1053	0.2720	0.0981	–
McFadden pseudo-R-squared	Nonsocial	0	0.0688	0.0762	0.0614	0.0694	–
	Social	0	0.1681	0.1546	0.1066	0.1622	–
Balanced accuracy	–	–	0.5929	0.6138	0.3863^a^	0.5^a^	–

a Rescorla–Wagner and Fehr–Schmidt models were assumed to be, respectively nonsocial and social models in calculating their balanced accuracy.

### Learning models fitting procedure

We estimated four models (Table 1): social learning model, sustainable nonsocial learning model, RW model and FS model on a trial-by-trial basis (Daw, 2011). Additionally, we estimated the null model as a benchmark. All models were estimated individually to the behavior of each participant by maximum likelihood estimation. The likelihood functions were optimized using Matlab 9.2 (MathWorks, Natick) (see S.I., section D). In all cases, the estimated parameters were constrained to lie within [0,1] for learning rate (α), social comparison and sustainability weights θ_s_ and θ_n_, and advantageous (δ^+^) and disadvantageous (δ^−^) inequality coefficients; and within [0, inf] for the inverse temperature β. After fitting the models, the estimated parameter values were later used to generate a learning process according to the specific model, so that various learning variables (i.e. sRPE, nRPE, social comparison component and sustainability component) could be determined. The predicted learning process and the learning variables were then correlated with the neural activity through parametric regressors in the SPM design matrix.

## Results

### Behavioral results

**Fig. 3.**
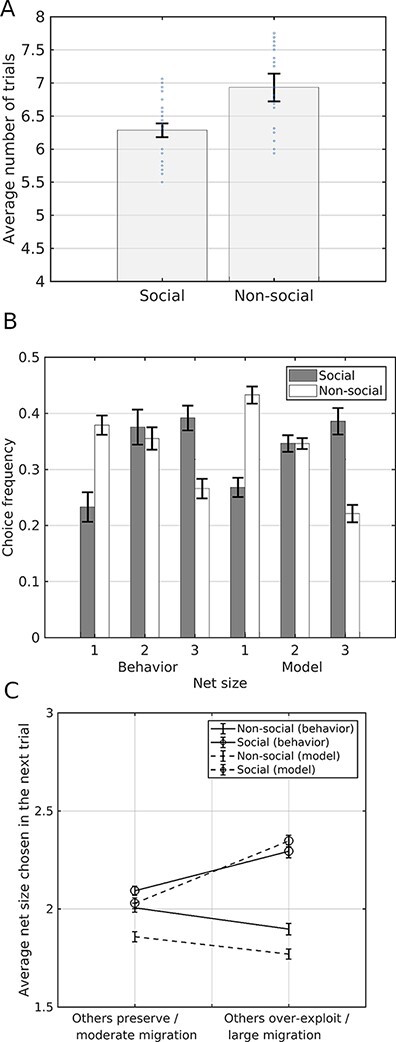
The experimental effects on resource depletion in the behavioral study and corresponding posterior predictive checks. Participants used the larger net size and depleted the resource faster in the social condition than in the private (nonsocial) condition, similar to the main fMRI study. (A) Mean number of trials per session in the two experimental conditions. The graph illustrates faster depletion of the resource in the social than in the nonsocial condition. Each session continued as long as the resource was sustained, with a maximum of eight trials. (B) Mean net size decision and posterior predictive check. Participants decided to take one fish more often in the social than in the private condition. The opposite was true for the largest net size of three fish. (C) Mean fish catch decision (in next trials) following resource depletion or preservation due to behavior of others or migration and posterior predictive check. After the depletion of the resource by others (social condition), participants also increased the fish catch in the next trial, whereas in the private (nonsocial) condition, an analogous reduction of the fish stock triggered instead a resource preservation reaction. Moderate migration/others preserve denotes a decrease of 2–4 fish; large migration/others exploit denotes a decrease of six fish. Error bars denote s.e.m.

Participants depleted the CPR faster in the social than in the nonsocial condition: average number of 6.28 (s.d. = 0.52) trials in the social condition as compared to an average number of 6.93 (SD = 1.06) trials in the nonsocial condition; two-sample *t*-test: *t*(48) = 2.703, *P *= 0.0095. Furthermore, different styles of fishing in the two conditions were indicated by an interaction of *Net Size* (1, 2 or 3 fish)* × Condition* (social, nonsocial), *F*(2.45) = 15.41, *P *= 0.0001. The participants used the smallest net size more often in the nonsocial condition than in the social condition (Figure 3), whereas they used the largest net size more often in the social condition than in the nonsocial condition. Crucially, in the social condition, after others overexploited the fish resource (six fish extracted in total), the participants in return also overexploited the resource in the next trial. In contrast, in the nonsocial condition a similar reduction of the fish stock (six fish migrated) triggered a trend toward resource preservation. This observation was supported by an interaction of *Resource Reduction* (small = 1, large = 3) × *Condition, F*(2.45) = 9.67, *P *= 0.003 (Figure 3C).

Modeling of the behavioral results in the social condition further supported the role of social comparisons in overharvesting of CPR. Perceived depletion of CPR by others facilitated overharvesting behavior in subsequent trials through social comparison: the individual weights of the model given to the social comparison correlated with the relative increase of harvesting in the trials following CPR depletion (i.e. mean selected net size in the trials following resource depletion by others minus mean selected net size in the trials following resource preservation by others; *r *= 0.49, *P *= 0.015, *n *= 24).

Next, we assessed the interaction effect between social and nonsocial models and conditions (Figure 2). The choice sensitivity parameter values were fairly homogeneous across both subjects and model type fit (β ∼ 1.5, Figure S3), whereas learning rates varied greatly across both participants and model types (Figure S3). We also used a linear mixed-effects model (LME) to test the effect of *Model Type* (four model types) and *Condition* (nonsocial, social) on BIC score with the participant as a random effect grouping factor (Table 3), with random intercepts to account for the unobserved heterogeneity due to sampling subjects from a population, that is, to allow generalizing statistical inference to the population level. Random slopes were not included because the variability in the model type and condition predictors across participants was too low to yield meaningful random-effects estimates. The LME was fit with the Matlab function fitlme, which implements restricted maximum likelihood with a trust region based on a quasi-Newton optimizer. The LME coefficients corresponding to the interaction between model types and conditions were all negative except for RW, suggesting that BIC scores were lower in the social condition for the other models (Table 3); however, this is likely due to the lower number of trials in the social condition (Figure 3A). To further examine interactions, an ANOVA was performed using the Satterthwaite approximation to the effective degrees of freedom afforded by the LME (Table 3) to test the effect of *Model Type* and *Condition*. The *F*-statistics and *P*-values of the *Model Type* main effect and the interaction term were *F*(4192) = 3.58, *P* = 7.7e-3 and *F*(4192) = 5.86, *P* = 1.79e-4, respectively. Therefore, the learning models’ scores were congruent with the observed behavior across social and nonsocial conditions. This agreed with the social comparison and sustainability weight estimates: the social component was higher in the social condition and the sustainability weight was higher in the nonsocial condition (Figure S3). To confirm this result we sought to ascertain directly whether the social model fit the social (and the nonsocial model fit the nonsocial) group data better in a mixed ANOVA (which does not rely on the Satterthwaite approximation of the LME model analysis) with participant as grouping random effects to test the interaction between *Model Type* and *Condition* (Table 4, Figure 3). Mauchly’s test reported no violation of the sphericity assumption. The interaction term was larger than zero (*F*(1,48) = 8.98, *P* = 4.3e-3), confirming the congruency between social and nonsocial models and conditions. Thus, the social learning model fits behavioral data during the social condition better than the sustainable nonsocial learning model, whereas the sustainable nonsocial learning model fits behavioral data in the nonsocial condition better than the social learning model. Finally, we computed aggregate BIC scores within each condition for each model. The BIC-based Bayes factors obtained by pairwise subtracting BIC score pairwise differences of more than 2 are considered as positive evidence, and of more than 10 as very strong evidence (Kass and Raftery, 1995). According to this measure, the social learning model was best in the social condition, and the sustainable nonsocial model fared the best in the nonsocial condition but not significantly better than the RW model—which is also a sort of nonsocial learning model. To assess to what extent sRPE and nRPE are dissociable, we also fitted a hybrid model combining both social and nonsocial RPEs in both social and nonsocial conditions. This hybrid model comprised a continuous parameter ξ ∈ [0, 1] that weighted the sRPE and nRPE (SocComp and SustComp, respectively, see equations (3 )and (6) such that for ξ = 0 it degenerates into the social model, and for ξ = 1 it becomes the nonsocial model. The estimated ξ was ξ = 0.26 ± .07 in the social condition was and ξ = 0.61 ± .06 in the nonsocial condition (where ± denotes s.e.m.), thus showing that parameter estimation within the hybrid model was sensitive to the type of RPE. However, the BIC scores of the hybrid model showed that its fit was worse than its social and nonsocial competitors (social: 5269.5, nonsocial: 6159.2, cf. Table 2). To sum up, participants depleted CPR faster in the social than in the nonsocial condition, and this overexploitation could be explained by a learning mechanism modulated by social comparison in the social condition. The model recovery analysis results (Table 5) show that both the social and nonsocial artificial players were identified (as measured by BIC scores, not shown) to belong to their actual class, more often than to any other class. The associated fitted parameters can be found in Figure S3 in S.I. The parameter ranges are roughly in agreement with the behavioral fits, but with a substantially larger variance, which is expected. The poor concordance for the FS and RW models is likely due to the lower estimation precision associated with worse goodness of fit. In brief, model recovery analysis results that models and parameters can be in principle recovered by using a rule, whereby the model with the lowest BIC score is selected.

**Table 3.** LME model fit with BIC as response variable; model type and condition with their interaction as fixed effects predictors and random intercepts (grouped by subject) predictors (BIC ∼ 1 + ModelTypeFactor * ConditionFactor + (1 | SubjectFactor), df = 240 for all predictors. Condition is a dummy variable denoting 1 for the social condition and 0 for the nonsocial condition. ModelType is a factor with 5 levels: the null model (reference level) and the four displayed models. Only fixed effects coefficients are shown

Predictor	*ß*	*SE*	*t*	*P*	Confidence int.
Intercept	248.33	6.48	38.3	∼0	[235, 261]
Social	−8.06	3.14	−2.56	0.011	[−14.2, −1.86
Nonsocial	−9.56	3.14	−3.04	2.63e-3	[−15.8, −3.36]
Rescorla–Wagner	−9.52	3.14	−3.03	2.72e-3	[−15.7, −3.33
Fehr–Schmidt	2.16	3.14	0.688	0.492	[−4.03, 8.36]
Condition	−22.8	9.34	−2.44	0.015	[−41.2, −4.40
Social × Condition	−13.9	4.53	−3.07	2.4e-3	[−22.9, −4.98
Nonsocial × Condition	−3.29	4.53	−0.724	0.469	[−12.2, 5.65]
Rescorla–Wagner × Condition	−9.46	4.53	−2.08	0.038	[−18.4, −0.515
Fehr–Schmidt × Condition	−14.6	4.53	−3.21	1.5e-3	[−23.5, −5.63]

**Table 4.** Mixed ANOVA testing the effect of the factors model type (social and nonsocial) and sociality condition on BIC score

Mixed ANOVA	SumSq	DF	MeanSq	F	*P*	p-GG^a^	p-HF^a^	p-LB^a^
(Intercept): ModelType	0.0804	1	0.0803	4.2e-4	0.984	0.984	0.984	0.984
Condition × ModelType	1707.2	1	1707.2	8.98	4.32e-3	4.32e-3	4.32e-3	4.32e-3
Error(ModelType)	9129.1	48	190.19					

a : *P*-values based on corrected degrees of freedom; GG: Greenhouse-Geisser correction; HF: Huynh–Feldt correction; LB: lower bound correction. SumSq: sum of squares. DF: degrees of freedom. MeanSq: mean SumSq per DF.

**Table 5.** Model recovery analysis. Fraction of runs (over a total of 50) simulating the social and nonsocial learning models (in rows) that were identified as belonging to each of the learning models described in the main text (in columns)

	Social	Nonsocial	Rescorla–Wagner	Fehr–Schmidt
Social	0.38	0.34	0.14	0.14
Nonsocial	0.26	0.34	0.26	0.14

### Neuroimaging results

**Fig. 4.**
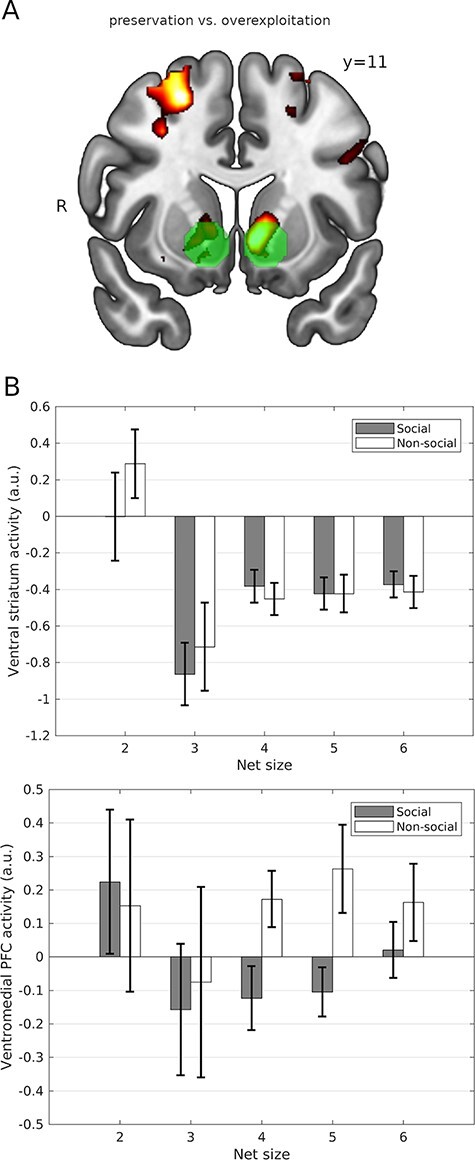
General effects of resource depletion: neural response to sharp resource depletion (six fish taken out as a result of migration or overharvesting by others) *vs* neural response to resource preservation (2–4 fish removed). (A) Map of deactivations (red-yellow) induced by resource depletion in both experimental conditions, and ventral striatum ROI (translucent green). (B) Ventral striatal and ventromedial prefrontal cortex activation evoked by overexploitation/preservation (social condition, *n *= 22) and by large/moderate migration (nonsocial condition, *n *= 24). Error bars denote s.e.m. Map thresholded at *P *< 0.001 uncorrected.

**Fig. 5.**
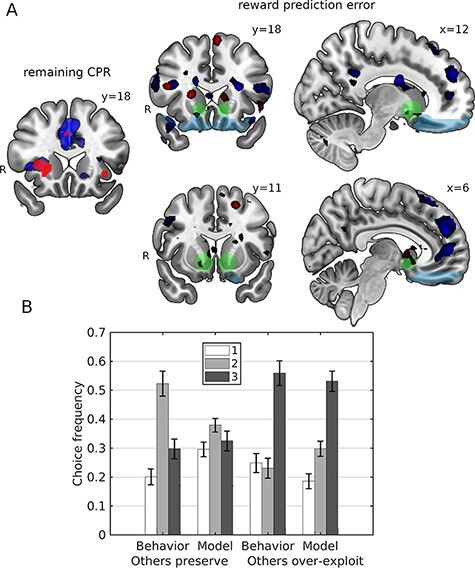
Neural activity involved in monitoring and managing CPR exploitation. (A) The role of the ventral striatum in CPR exploitation monitoring and learning. Left: neural deactivations associated with the size of the remaining CPR, indicating that activity was parametrically modulated by the change of the CPR size trial-by-trial in social (red) and nonsocial (blue) groups. Right: activity associated with learning signals: social (red) and nonsocial (blue) reward prediction errors; ventral striatum ROI (translucent green) and vmPFC ROI (translucent light blue). (B) Posterior predictive checks. Left: average probability of choosing net size (1, 2 or 3) after sharp CPR depletion by others in the previous trial (six fish taken out by others) matched the observed frequency of the overharvesting (choosing the largest net size). Right: the model predicted the tendency to preserve CPR after conspecifics also chose to preserve in the previous trial (four fish or fewer taken out). Model prediction refers to the probabilities estimated with the fitted models, whereas Behavior indicates subjects’ choice frequencies. Maps are thresholded at *P *< 0.005, uncorrected.

A sharp decrease of the CPR (extraction of six fish due to overexploitation by others or to extensive migration) was associated with ventral striatum deactivation more strongly than a moderate CPR decrease (extraction of four or fewer fish) in both conditions (Figure 4A, Table S2). A mixed ANOVA test (*Subject* as random factor and *Condition* and *CPR decrease* as fixed factors) yielded a main effect for *CPR decrease*: F(1,86) = 10.06, *P* = 0.002). In the social condition, ventral striatum activations were smaller than in the nonsocial counterpart (factor *Condition*: F(1,86) = 5.62, *P* = 0.019 in an analogous mixed ANOVA test). However, we could not find in the social condition statistical evidence that overexploitation by others evokes stronger deactivation of the ventral striatum than the similar large migration in the nonsocial condition (Figure 4B, top), according to a mixed ANOVA test interaction term F(1,86) = 2.44, *P* = 0.121 (*Subject* as random factor, and *Condition* and *CPR decrease* as fixed factors). However, it is worth noting here that the total number of small net size trials in pre-recorded data was much smaller than for large net size trials (2: 3.4%, 3: 2.7%; 4: 34.9%; 5: 25.3%; 6: 33.7%), so the actual (within-subject) uncertainty associated to the bars corresponding to 2 and 3 is much larger than for 4, 5 and 6. However, there was some evidence for a dissociation in the vmPFC activation sign for social *vs* social conditions during large net size trials (Figure 4B, bottom). To further test the hypothesis that the ventral striatum differently monitors the resource changes in social and nonsocial contexts we conducted a more detailed parametric analysis. Using the total number of fish remaining in the lake in every trial as the modulation parameter, we found an effect of the total resource change on the activity of the ventral striatum: activity of the ventral striatum negatively correlated with CPR depletion (remaining CPR, Figure 5A left). As shown in Figure 5B, the posterior predictive check suggests that the social learning model predicted the overexploitation of CPR. This was supported by posterior predictive checks for choice frequencies (Figure 3B and C). Using parametric fMRI analyses, we investigated the modulation of the ventral striatum (Figure 5A right) and vmPFC activity by different versions of RPE (Table 4). Social RPE (sRPE) was defined as the RPE in the social learning model, whereas nonsocial RPE (nRPE) was defined by the sustainable nonsocial learning model. ROI-average analyses indicated that sRPE modulated activity of the ventral striatum in the social group (df = 21, *t* = 2.19, *P* = 0.040) but there was no evidence that it did in the nonsocial group (df = 23, *t* = 1.52, *P* = 0.14). There was some evidence for nRPE modulating activity in the ventral striatum in the nonsocial group (*t* = 2.04, *P* = 0.053), and for a neural dissociation in the sense that sRPE correlated with striatal activity more in the social than in the nonsocial group (*t* = 1.97, *P* = 0.062), and nRPE correlated with striatal activity more in the nonsocial than in the social group (*t* = 2.79, *P* = 0.010); this would suggest that striatal dopaminergic regions differentially monitor resources in the social and nonsocial conditions. Finally, we found no evidence in favor of nRPE modulating activity in vmPFC in both social (*t* = 1.58, *P* = 0.128) and nonsocial groups (*t* = 1.80, *P* = 0.085), and likewise for sRPE (*t* = 1.32, *P* = 0.20 for social group; *t* = 1.06, *P* = 0.29 for nonsocial).

## Discussion

The current study explores the differences of how people deal with a private good as compared to a common/public good. The results of our study indicate that during the CPR task the ventral striatum encodes opposite harvesting strategies: relative deactivation of the ventral striatum in response to resource depletion correlates positively with participants’ attempts to preserve their own private resources and correlates negatively with their attempts to preserve the CPR. The ventral striatum receives dopamine projections from the midbrain and is activated by a wide range of rewarding stimuli, from foods, odors and drugs to beautiful faces (Breiter *et al.*, 1997; Aharon *et al.*, 2001; Gottfried *et al.*, 2002; O’Doherty *et al.*, 2004). The activity of the ventral striatum was also associated with social comparison of collected rewards (Fliessbach *et al.*, 2007; for a meta-analysis, see Luo *et al.*, 2018), voluntarily donations (Moll *et al.*, 2006; Harbaugh *et al.*, 2007), mutual cooperation (Rilling *et al.*, 2002, 2004) and even the punishment of others who have previously behaved unfairly (de Quervain *et al.*, 2004; Singer *et al.*, 2006).

Previous research shows that the ventral striatum exhibits more activity when players choose cooperation following a cooperative choice by their partners in the previous round of the iterated Prisoner’s Dilemma (Rilling *et al.*, 2002). Furthermore, people with a higher desire for revenge against unfair partners exhibited activation in the ventral striatum (Singer *et al.*, 2006). Participants who made more costly donations to real charitable organizations also exhibited more activity in the striatum (Moll *et al.*, 2006). Overall, our results are consistent with the previous studies indicating the critical role of the ventral striatum in cooperative behavior.

We develop a computational learning model that allows us to suggest a neurocognitive explanation for CPR depletion. The model uses a social RPE signal governing the learning updating process, which correlates with ventral striatum activity. This might indicate that the striatum harbors the RPE signal where the reward of an outcome is composed of the person’s own monetary reward and a comparison of the person’s own outcome with the outcomes of others. In contrast, in the nonsocial condition, the classical RW model matched the sustainable nonsocial learning model, which does not bear out the existence of a specialized learning mechanism in the nonsocial condition. The goodness of fit of computational models (Figure 2) shows that the social condition fits (as indexed by BIC scores, but also by log-likelihood) have higher variance across subjects, with lower means and medians, which suggests that on average participants learned less in the nonsocial condition (at least in terms of incremental adaptive learning). This is also in agreement with the stronger modulation of ventral striatum activity in response to perceived decreases of CPR, in the social condition than in the nonsocial condition. The strong reactivity of the ventral striatum in the social condition is to be expected in its role of integrating social values because a scarce resource shared by people is much more likely to be depleted than in the nonsocial situation. Thus, the social model predicts the enhanced selfish behavior of humans under a scarcity of resources. Our fMRI results indicate that the dopamine system is involved in social comparisons and generates a negative prediction error when a person receives less than the competitors and a positive prediction error when she receives more than the competitors. Thus, ventral striatum activity not only monitors outcomes (resource depletion) but also integrates outcomes into the specific social context. Perhaps the dual nature of the reward-monitoring activity explains our observation that behavioral tendencies underlying competitive depletion of resources are differentially encoded in the activity of the ventral striatum in social and nonsocial contexts. To sum up, cognitive modeling demonstrated that the brain may resort to distinct strategies depending on the social framing of the task and that this framing modulates neural activity accordingly. Overall, our results are consistent with the hypothesis that social rewards and social preferences are represented in the ventral striatum similarly to primary or monetary rewards (Montague and Berns, 2002; Fehr and Camerer, 2007).

The conclusions of our study have some limitations. Similar to other standard behavioral games that allow unambiguous inferences, participants in our study act fully anonymously and independently of each other. They are given no opportunity to discuss the situation or to change the institutional rules. However, these opportunities might exist in real-life situations and could also provide a way of avoiding the depletion of the resource (Ostrom, 1990). Although participants had reasons to believe that they could interact with opponents in a temporally delayed fashion, the ecological validity of this approach has not been thoroughly tested. More studies are needed to verify our neuroimaging results using real interaction play and to investigate the strategic aspects of CPR depletion.

Additionally, our model of social comparison assumes that receiving more than the competitors is perceived as a positive reward. Although on average this assumption leads to a good description of the overall results, there might be an individual difference in social preferences, which the model cannot account for. Follow-up studies will help to examine alternative interpretations of the activity of the ventral striatum observed in our study, e.g. as a neural correlate of the perceived violation of warm glow preferences (Andreoni, 1990; Harbaugh *et al.*, 2007) or of the altruistic norm by others.

Neuroimaging studies have demonstrated that cooperation consistently activates not only reward systems such as the vmPFC and ventral striatum, but also medial prefrontal cortex, temporoparietal junction (TPJ), and superior temporal sulcus (McCabe *et al.*, 2001; Rilling *et al.*, 2002; Decety *et al.*, 2004; King-Casas *et al.*, 2005; Elliott *et al.*, 2006). Furthermore, competition may also activate inferior frontal gyrus and dorsolateral prefrontal cortex (Decety *et al.*, 2004; Lissek *et al.*, 2008; Halko *et al.*, 2009; Lee *et al.*, 2018a). Some of these regions are likely to subserve mentalization, the ability to understand one’s own or others’ mental states as causes of behavior. TPJ has been implicated in self-other distinction and theory of mind (Saxe and Kanwisher, 2003; Samson *et al.*, 2004; Frith and Frith, 2012), whereas dorsomedial PFC has been similarly associated with mentalization (Hampton *et al.*, 2008; Coricelli and Nagel, 2009; Lee *et al.*, 2011), altruism (Waytz *et al.*, 2012) and morality (Bzdok *et al.*, 2012). The reason TPJ and dmPFC were not considered is that our study focused on the modulatory influences of social factors on behavior, through value learning. In particular, because our study was designed to probe (i.e. maximize test sensitivity for) the modulation of learning processes under different social contexts, we were not able to assess the putative involvement of mentalizing areas. Studies of the theory of mind typically investigate belief attribution by presenting concocted scenarios evoking mentalization and querying participants about the beliefs of others (Saxe and Kanwisher, 2003; Lee *et al.*, 2011), or use a controlled design that allows specifying some index of mentalization such as the depth of strategic reasoning (Coricelli and Nagel, 2009). Such schemes were troublesome to incorporate simultaneously in our study because treatments were between subjects, and participants’ responses were limited to numerical choices. Further studies and different behavioral paradigms will be needed to identify the role of these regions in competitive overexploitation of common resources in different social contexts.

For a long time, behavioral economics focused on examining factors that favor CPR preservation, including the best possible rules, institutions and communication (Ostrom, 1990). Social psychologists searched for psychological determinants of individual cooperative *vs* self-interested behavior in commons-dilemma situations (Messick *et al.*, 1983). Our results show that the context of a shared resource *vs* a private resource (with similar control over the resources in both contexts) modulates the neural activity of the ventral striatum—a brain area strongly associated with the valuation of outcomes. Overall, the notion of the neurobiological underpinnings of resource overexploitation could help us to develop efficient boundary rules and a better understanding of global commons governance.

## Supplementary Material

nsac008_Supp

## Data Availability

Experimental behavioral logs and MRI data are available on the Open Science Framework website (https://osf.io/3zepd/) under the CC0 1.0 Universal license. Source code for preprocessing data and fitting behavioral models in MATLAB is available on the hosting service GitHub (https://github.com/mmartinezsaito/fish-cpr) under the MIT license.
