# Supplementary material for: Mine or ours? Neural basis of the exploitation of common-pool resources
Source: Soc Cogn Affect Neurosci. 2022 Feb 1;17(9):837–49. doi: 10.1093/scan/nsac008 (PMC9433840; doi:10.1093/scan/nsac008)
Supplement: nsac008_Supp [file nsac008_supp.zip › Supplementary.docx]

**Supplementary Information for “Mine or ours? Neural basis of the exploitation of common-pool resources”**

Mario Martinez-Saito, Sandra Andraszewicz, Vasily Klucharev, Jörg Rieskamp

**Table of contents**

| A. Pilot behavioral study | 2 |
| --- | --- |
| B. Behavioral study | 3 |
| C. Game-theoretical analysis of the CPR task | 4 |
| D. Optimization algorithms details | 7 |
| E. PPI analysis | 8 |
| Figures | 9 |
| Tables | 13 |
| References | 16 |

**A. Pilot behavioral study.**

The pilot behavioral study consisted of a common pool resource (CPR) experiment. The aim of this experiment was to test the CPR paradigm and to collect behavioral data for the follow-up behavioral and imaging studies.

***Participants*.** In each experimental session, three participants played with each other simultaneously while dealing with a CPR. The participants (N=24, aged 18–28 years, mean 21.8 years, 9 females) performed the task simultaneously (Figure 1, main text). Participants performed 20 sessions (8 trials per session). The tasks were performed in groups of six participants and in separate cubicles to ensure anonymity.

***Experiment*.** Participants were informed that they were participating in a study investigating decision making. The task design was the same as in the main experiment, except that there was only a social condition and play was simultaneous and *interactive*. The experiment started with a short training session. The task was programmed with the software z-Tree (Fischbacher U., 2007).

***Results*.** Overall, the participants did not follow the game-theoretical prediction of completely self-interested people who would always select the largest net size for all trials in the game. Nevertheless the participants over-harvested and depleted the CPR: on average 58.7% (s.d.=32.5) of sessions were completed before the 8^th^ trial, which indicated over-harvesting behavior (mean number of trials in a session = 7.4). The average selected net size (net size = 2.3) was significantly higher than the “sustainable” size of the net (net size=2), *t*(1,23)=5.73, *p*=8e-6. Two highly competitive participants (the average net size=2.6 and 2.7) were selected for the fMRI version of the study and their behavioral results were used in the social and non-social conditions. This was done because the study’s goal is assessing the impact of resource depletion in a common goods dilemma: cooperating players would preclude testing the research question. This raises the question of whether the validity of the main behavioral results is restricted to participants exposed to highly competitive players. As reported above and in the next section, participants depleted the CPR faster in the behavioral study (average of ~6.2 trials before depletion in the social condition, section B) than in the pilot behavioral study (~7.4 trials, section A). We surmise that the reason lies at least in part in the selection of competitive players from the pilot behavioral. However, the pilot study shows that more than half of the participants were highly competitive, in the sense that they depleted consistently the CPR, so the choice of competitive participants is unlikely to belie the ecological validity of the experiment. In an alternative scenario where participants faced mostly cooperative players, we expect that the neural signatures of prediction errors under social and non-social conditions would become undissociable, and perhaps involvement of theory of mind regions such as the temporoparietal junction.

**B. Behavioral study.**

The goal of this behavioral study was to examine how people deal with a social resource as compared to a non-social resource. We used a modified version of the CPR task from the pilot behavioral study (section A). The experiment was identical to the fMRI version design, but it was conducted in a behavioral laboratory.

***Participants*.** We invited thirty-seven healthy students to test the CPR task. To avoid any demand effects and suspicion toward the two different (but structurally identical) conditions, we implemented a between-subjects design: Participants were randomly assigned to the social or non-social condition of the CPR task (with N=19 for the social and N=18 for the non-social condition). Overall, they played 16 sessions (8 trials per session).

***Experiment*.** The design was as in the pilot behavioral experiment (section A) but with two conditions. In every trial, participants decided between three possible net sizes for fishing one, two, or three fish. In the social version of the experiment (social condition), two other participants (pre-recorded from the pilot behavioral study) also decided between the three net sizes. In the non-social version of the experiment (non-social condition), the same number of fish “migrated” to two neighboring lakes. Importantly, the change of the resources due to the two other pre-recorded participants or the "migration" to the two neighboring lakes was identical in both conditions.

***Results*.** Similar to the fMRI experiment, participants depleted the resource of fish significantly faster in the social condition than in the non-social condition (the mean number of trials in the social condition was 6.24 versus 7.00 in the non-social condition, *t*(1,35)=3.30, *p*=.002). The average selected net size was significantly larger in the social condition (2.09) than in the non-social condition (1.85), *t*(1,35)=2.25, *p*=.015. We observed different styles of behavior in the two conditions as indicated by a significant interaction *Net size (one, two, three fish*) × *Condition* (*non-social, social*): *F*(2.34)=3.99, *p*=.028. Participants used more often the smallest net size in the non-social condition than in the social condition and the largest net size was selected more often in the social condition than in the non-social one. Similar to the results in the fMRI study, in the social condition, after the over-exploitation of the fish resource by others (6 fish drawn out by others), participants then also over-exploited the resource in the next trial. However, in the non-social condition, a similar reduction of the fish stock (6 fish migrated) led to resource preservation. This observation was supported by a significant interaction *Resource reduction (small, large)* x *Condition,* *F*(1.35)=7.44, *p*=.010. The results were later replicated in the behavioral results of the fMRI study reported in the main text, providing independent additional evidence for the observed results. The distribution of resource depletion used in the fMRI experiment (for both social and non-social conditions) was calculated from two participants in the behavioral study and was as follows: [3.35 2.66 34.58 25.29 34.12] (in %) over net sizes [2 3 4 5 6].

**C. Game-theoretical analysis of the CPR task**

What is the game-theoretical solution for the fishing game when assuming only self-interested and rational (i.e. payoff-maximizing) players? In the CPR task, the solution can be easily determined by backward induction. The task has a finite number of trials which are common knowledge to all players. Therefore, it is clear that in the very last trial, it is best for everyone to choose the largest net size to maximize payoffs. Given this behavior, it is also rational to choose the largest net size in the second-last trial, and so on. Therefore, the game-theoretical solution is to choose the largest net size in all trials of the task.

How should a self-interested player behave in the non-social situation (non-social condition), in which no other players are involved? Here the solution depends on a person’s belief about the amount of fish that migrates to the two other lakes. If a person believes that the migration rate is low, then the person should choose the largest net size all the time. In contrast, if the player believes that the migration rate is high, it can be payoff-maximizing to choose a small net size to sustain the resource to allow for future consumption. However, the optimal behavior will depend on the specific beliefs about the migration rate. When assuming uniform priors of players’ beliefs about the migration rate, it can be predicted that the consumption rate should be lower in the non-social condition than in the social condition, which is in line with the behavioral findings.

More specifically, we determined the optimal behavioral strategy for the game in the non-social condition given different beliefs about the migration rate. The migration to the first lake is represented by $L_{t}^{1}$, the migration to the second lake by $L_{t}^{2}$, and its sum represents the total migration *L_t_* for trial *t*. The beliefs about migration can be represented by the probability with which a player believes that the particular migration rate occurs, that is $\text{Pr}\left( L_{t}^{1} \right)$ and $\text{Pr}\left( L_{t}^{2} \right)$ (note the migration to each lake is discrete and ranges between 1 and 3 fish). We examined three different assumptions about the players’ beliefs. First, we assumed that all three possible migration rates for each lake were constant and equally likely (Belief 1). Second, we assumed that the players’ beliefs about the different migration rates would reflect the average migration observed in the whole task (Belief 2; i.e. if a migration of 2 fish to one lake occurred in half of all trials and sessions, the probability would be .50). Third, we assumed that a player would start with an initial belief that every migration rate would be equally likely (Belief 3).

After completing the first session, this belief is updated according to the observed migration rates in each trial. To update the belief after the completion of session *S* we determine:

$\text{P}\left( L_{t}^{i} \right)_{S}\text{ = }\frac{1+\sum f_{j}\left( L_{t}^{i} \right)}{S+1}$, (1)

where *L* represents the three possible migration rates of 1, 2, or 3 to one of the two lakes *i*, and *f*(.) represents an indicator function that takes a value of 1 if the particular migration occurred in the trial *t* and a value of 0 otherwise.

Given the players’ beliefs about the possible migration rates, we determined the optimal strategy for the whole task, specifying the chosen net size for each trial of the task using a dynamic programming approach. For the very last trial (i.e. the eighth trial) it is possible to determine the expected payoff of choosing each of the three possible net sizes. The expected payoff in the eighth trial depends on the chosen net size, the remaining number of fish, and the probability of the different migrations rates. It can be easily seen that the highest expected payoff will always result from choosing the largest net size in the last trial. From this eighth trial we determine the optimal strategy in the seventh trial. Here, a complete strategy specifies the chosen net size for the seventh trial and the eighth trial. The expected payoff for the strategies in trial seven depends on the payoff in the seventh and the eighth trial. The payoff in the eighth trial depends on the remaining number of fish, which depends on the chosen net size and migration in the seventh trial. Thus, the chosen net size in the seventh trial does not only influence the immediate payoff but also the possible payoffs in the eighth trial. Following this approach, the optimal strategy can be determined for the sixth trial, where the expected payoffs of all possible strategies depend on the chosen net size in the sixth trial and the chosen net sized in the seventh and eighth trial. Following this backward induction one can determine the overall best strategy for the whole task starting in the very first trial.

Mathematically, the expected payoff given a player’s strategy for the whole task is calculated as the total payoff that can be obtained in the task multiplied by the probability of obtaining this payoff given a particular strategy:

$\text{E}\left[ \text{Payoff|Strategy} \right]\text{ = }\sum\text{payoff}\cdot\text{P}\left( \text{Payoff=payoff|Strategy} \right)$ (2)

The probability of obtaining a specific total payoff depends on the strategy. On the one hand, the strategy defines the net size and affects the payoffs, but it also affects the development of the resource and thereby the size of the resource in subsequent trials.

The results of this analysis are illustrated in Figure S1. When assuming that all migration rates are equally likely, then according to the best strategy, one should choose a net size of 1 in the very first trial, increase the net size to 2 in trial two to four and starting from trial five, one should always choose net size 3 (Belief 1 optimal solution). When assuming that the players would know the actual migration rates in all trials (which is unrealistic but interesting to set up as a benchmark), they should also choose net size 1 in trial 2, and net size 2 for trials two to four, and always a net size of 3 from trial five onwards (Belief 2 optimal solution). Finally, when assuming equal priors for the first trial that are updated on the observed migration rates, then it is optimal to choose net size 1 for trial 1 and to increase the net size for the following trials with a net size of 3 starting from trial six onwards (Belief 3 optimal solution). Overall, the analysis shows that given a variety of beliefs, the payoff maximizing strategy is not to choose the largest net size at the beginning of the task in the non-social condition, but to choose the largest net size at the end, starting at the sixth trial at the latest. Thus, according to this analysis, one would expect smaller net sizes in the non-social as compared to the social condition at the beginning of the task, which is consistent with the experimental findings. To sum up, independently of one’s belief, the game-theoretical optimal strategy in the non-social condition is to start from smaller net sizes and increase the net size towards the end of the game. The behavioral data was congruent with this strategy.

**D. Optimization algorithms details**

We used the interior-point and sequential quadratic programming methods of fmincon, a generic constrained nonlinear local optimizer; and six algorithms from the MATLAB Global Optimization Toolbox (Optimization Toolbox, Matlab 9.2, MathWorks, Natick, 2017): two global search algorithms which start the local solver fmincon from multiple starting points and sample multiple basins of attraction (GlobalSearch and MultiStart), pattern search optimization (patternsearch), particle swarm optimization (particleswarm), simulated annealing (simulannealbnd), and a genetic algorithm for search (ga). The local solver fmincon (73%) and the global optimizer GlobalSearch (20%) together accounted for 93% of the found best parameters (at the lowest minima).

**E. Instructions (translated from German)**

***Non-social condition***

In the following study you will be asked to make repeated decisions. Each decision will lead to a monetary payoff for you.

For the decision situation imagine that you are fishing in your **own private lake**. Your task is to collect as much fish as possible. Each fish that you collect will lead to a monetary payoff. You will encounter **16** sessions. Every session will consist of eight (8) rounds. In every round you have to decide between three possible net sizes for fishing. When you choose the smallest net size you will collect one fish, with the medium net size you will collect two fishes, and with the largest net size you will collect three fishes.

In addition fish can migrate to 2 neighboring lakes. Thus, the overall number of fishes in your lake will decrease by the total number of fish that is collected by you and by the number of migrated fish. Although the number of fishes in the lake decreases by fishing and migration it also grows naturally due to proliferation of fish. Therefore after you have collected fish the remaining number of fish in the lake is increased by 50% that is it is multiplied by 1,5, which gives the total number of fishes for the next round.

Total number of fish in the lake for the next round =

= (“Number of fishes in the lake” – “Number of fishes collected by you” – “Number of migrated fishes”) × **1.5**

The total number of fish in the lake is limited by 20 fishes, because of the limited space in the lake, i.e. the population does not grow above 20 fishes.

In case the remaining number of fishes in a round is zero, then this implies that no fish will exist for the next round and the whole session ends automatically and no further rounds in the session will take place (the next session will be started instead). Thus, if the session ends premature you will lose the possibility to collect further fish in this session. In case the total number of fish in the lake is lower than the net size you selected, you will get a number of fish proportional to your demand.


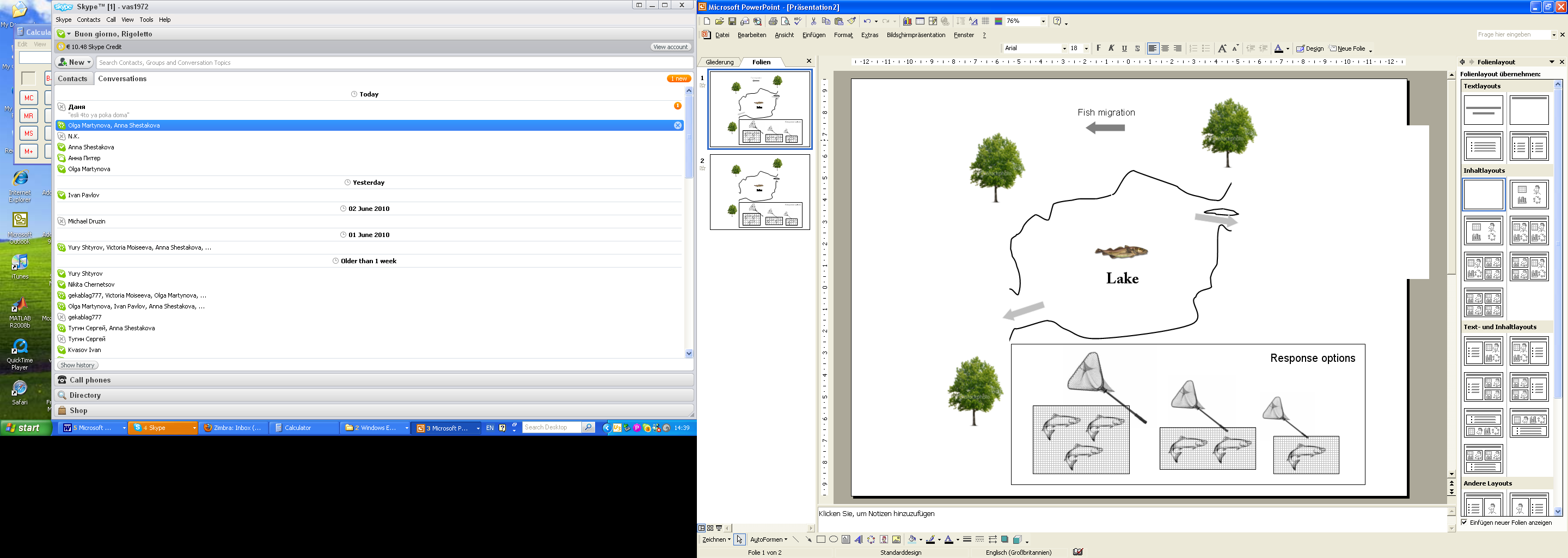


Each new session is indicated by the sign: “New Session”. All sessions start with the same initial number of fish in the lake, namely 18 fishes.

The following graph shows the different screen views that you will encounter during the experiment:


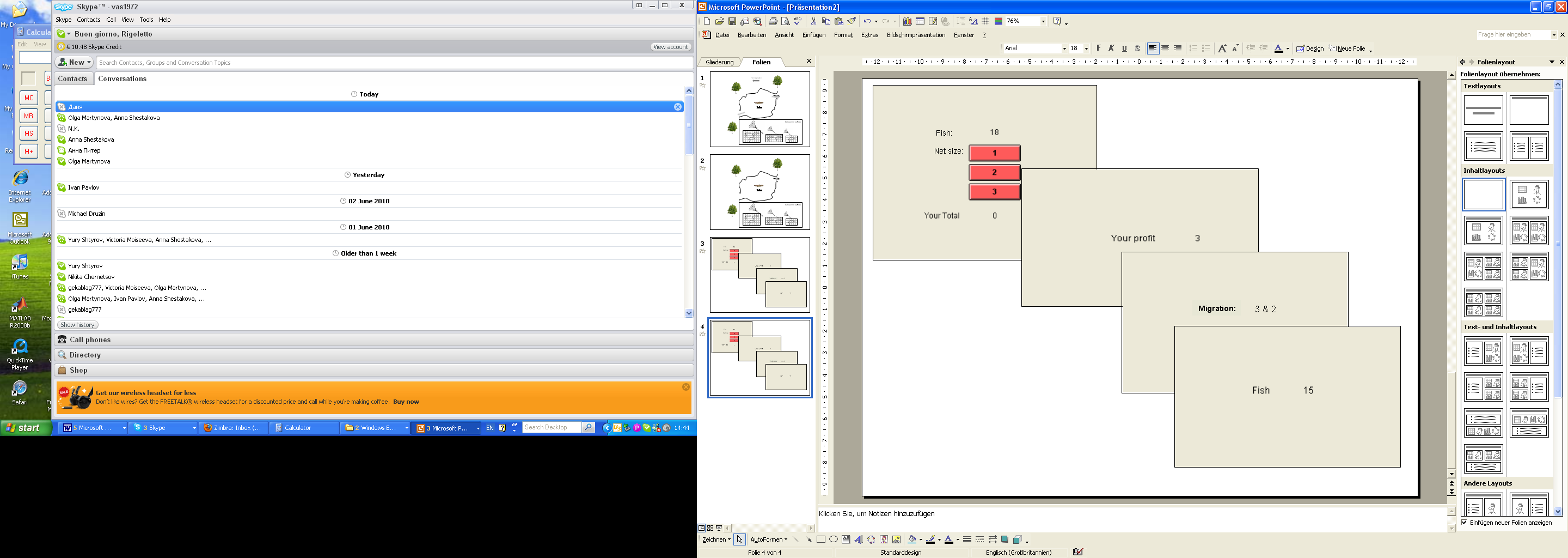


**1.** At the first screen you have to decide between the three possible net sizes (for 1, 2 or 3 fishes). In addition you will be provided with the current number of fishes in the lake. Furthermore you receive information about the total number of fishes that you had collected so far.

**2.** The second screen provides you with feedback about the number of fishes that you collected in the current round.

**3.** The third screen tells you the number of fishes migrated to 2 neighboring lakes.

**4**. Finally, the last screen informs you about the new total number of fishes that are available in the lake for the next round.

At the end of the each session you will be informed about your overall profit collected during the session.

To explain the decision situation in detail, consider the three following examples:

1. You always collect one (1) fish from the lake and one (1) fish migrated to 2 neighboring lakes (1 + 1 + 1 = 3 fishes) the fish population will increase. In this case you will have the possibility to collect always 1 fish in all rounds and the total number of fishes in the lake will increase over the sessions.
2. If you always collect two (2) fishes and two (2) fish migrated to 2 neighboring lakes from the lake (2 + 2 + 2 = 6 fishes) the fish population will stay constant. In this case you will have the possibility to collect always 2 fishes in all rounds and the total number of fishes in the lake will stay constant over the sessions.
3. If you always collect three (3) fishes and three (3) fish migrated to 2 neighboring lakes (3 + 3 + 3 = 9 fishes) the fish population will decrease. In this case you will not have the possibility to collect always 3 fishes in all rounds. Instead, after the fourth round the fish population has vanished and no further rounds will take place.

In general, whenever the sum of fishes collected by you and migrated is smaller than six the fish population will increase over the rounds. In contrast, whenever the sum of fishes collected by you and migrated is large than six the fish population will decrease over the rounds. Whenever the total number of fishes collected by you and migrated is equal to six the fish population will stay approximately constant over the rounds.

**Payment**

At the end of the experiment one of the 20 sessions will be chosen randomly. The number of fishes that you had collected during this session will determine your payoff. For each fish that you collected you will receive 0.25 Swiss Franks. In addition to this payment you will receive a compensation of 15 Franks in the form of a book certificate or 2 “Signatures.”

The experiment starts with a short training session.

Experiment lasts approximately 30 min.

Thanks a lot for taking part in our study.

***Social condition***

In the following study you will be asked to make repeated decisions. Each decision will lead to a monetary payoff for you. Which payoff occurs depends on your own decision and on the decision of two other persons with whom you interact. The decisions of two other players were pre-recorded. The payoffs of the other persons also depend on their decisions and on your decision (the other persons will be paid on the basis of the current experiment). All interaction will take place via the computer and is completely anonymous, that is neither you nor the other person will be informed about the identity of the persons taking part in this study.

For the decision situation imagine that you are fishing in a lake. Your task is to collect as much fish as possible. Each fish that you collect will lead to a monetary payoff. You will encounter **16** sessions. Every session will consist of eight (8) rounds. In every round you have to decide between three possible net sizes for fishing. When you choose the smallest net size you will collect one fish, with the medium net size you will collect two fishes, and with the largest net size you will collect three fishes. The other two persons will also decide between the three net sizes that collect the same number of fish. The total number of fish that is collected from the lake is simply defined as the sum of fish that all persons collect.

The overall number of fishes in the lake will decrease by the total number of fish that is collected by you and the other two persons. Although the number of fishes in the lake decreases by fishing it also grows naturally due to proliferation of fish and migration of fish. Therefore after every person has collected its fish the remaining number of fish in the lake is increased by 50% that is it is multiplied by 1,5, which gives the total number of fishes for the next round.

Total number of fish in the lake for the next round =

= (“Number of fishes in the lake” – “Number of fishes collected by the three persons”) × **1.5**

The total number of fish in the lake is limited by 20 fishes, because of the limited space in the lake, i.e. the population does not grow above 20 fishes.

In case the remaining number of fishes in a round is zero, then this implies that no fish will exist for the next round and the whole session ends automatically and no further rounds in the session will take place (the next session will be started instead). Thus, if the session ends premature you will lose the possibility to collect further fish in this session. In case the total number of fish is lower than the sum of net sizes of the three persons, each person will get a number of fish proportional to the total demand.


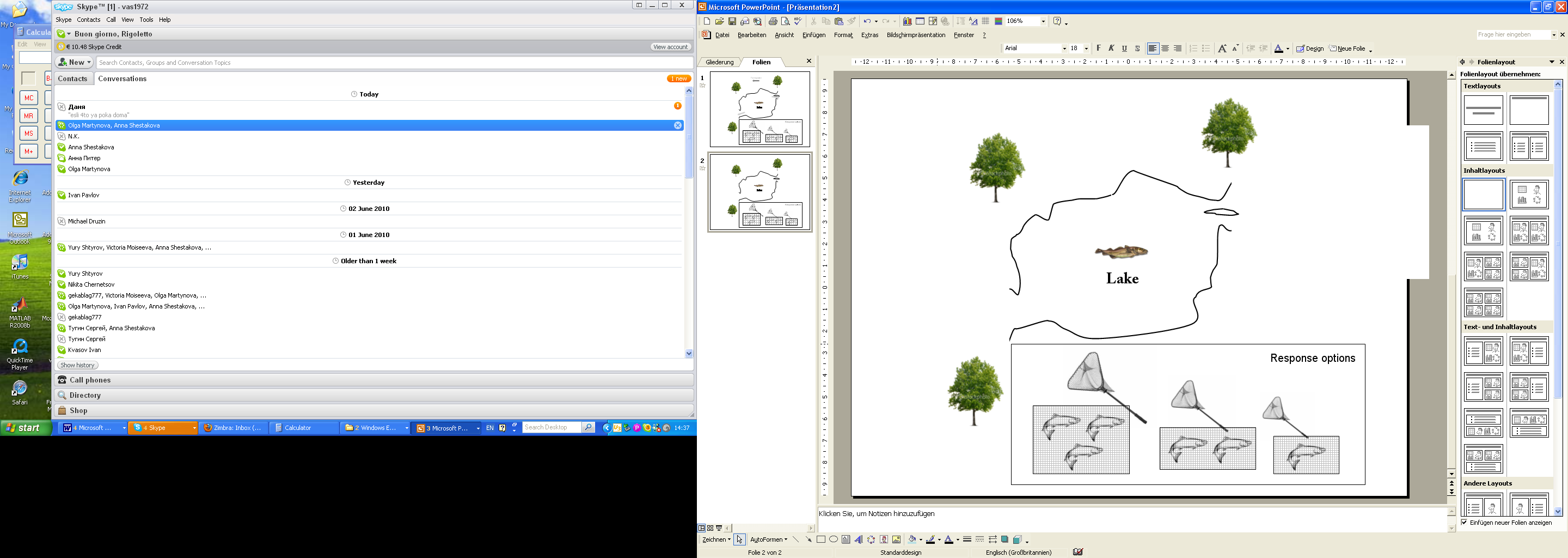


Each new session is indicated by the sign: “New Session”. All sessions start with the same initial number of fish in the lake, namely 18 fishes.

The following graph shows the different screen views that you will encounter during the experiment:


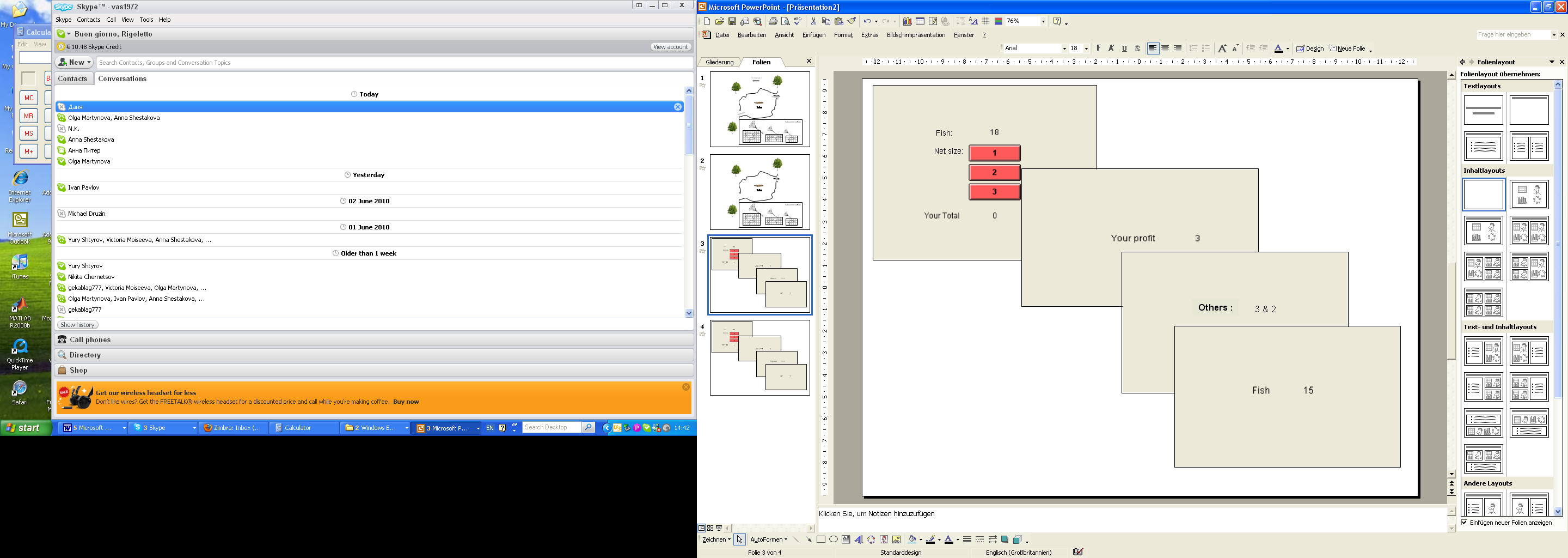


**1.** At the first screen you have to decide between the three possible net sizes (for 1, 2 or 3 fishes). In addition you will be provided with the current number of fishes in the lake. Furthermore you receive information about the total number of fishes that you had collected so far.

**2.** The second screen provides you with feedback about the number of fishes that you collected in the current round.

**3.** The third screen tells you the number of fishes the other two persons with whom you interact had collected.

**4.** Finally, the last screen informs you about the new total number of fishes that are available in the lake for the next round.

At the end of the each session you will be informed about your overall profit collected during the session.

To explain the decision situation in detail, consider the three following examples:

1. If all persons always collect one (1) fish from the lake (1 + 1 + 1 = 3 fishes) the fish population will increase. In this case all persons will have the possibility to collect always 1 fish in all rounds and the total number of fishes in the lake will increase over the sessions.
2. If all persons always collect two (2) fishes from the lake (2 + 2 + 2 = 6 fishes) the fish population will stay constant. In this case all persons will have the possibility to collect always 2 fishes in all rounds and the total number of fishes in the lake will stay constant over the sessions.
3. If all persons always collect three (3) fishes from the lake (3 + 3 + 3 = 9 fishes) the fish population will decrease. In this case all persons will not have the possibility to collect always 3 fishes in all rounds. Instead, after the fourth round the fish population has vanished and no further rounds will take place.

In general, whenever the total number of fishes collected by the three persons is smaller than six the fish population will increase over the rounds. In contrast, whenever the total number of fishes collected by the three persons is large than six the fish population will decrease over the rounds. Whenever the total number of fishes collected by the three persons is equal to six the fish population will stay approximately constant over the rounds.

**Payment**

At the end of the experiment one of the **16** sessions will be chosen randomly. The number of fishes that you had collected during this session will determine your payoff. For each fish that you collected you will receive 0.25 Swiss Franks. In addition to this payment you will receive a compensation of 15 Franks in the form of a book certificate or 2 “Signatures.”

All interaction will take place anonymously. The two persons with whom you interact are randomly selected from all persons who take part in the experiment. The experiment starts with a short training session.

Experiment lasts approximately 30 min.

Thanks a lot for taking part in our study.


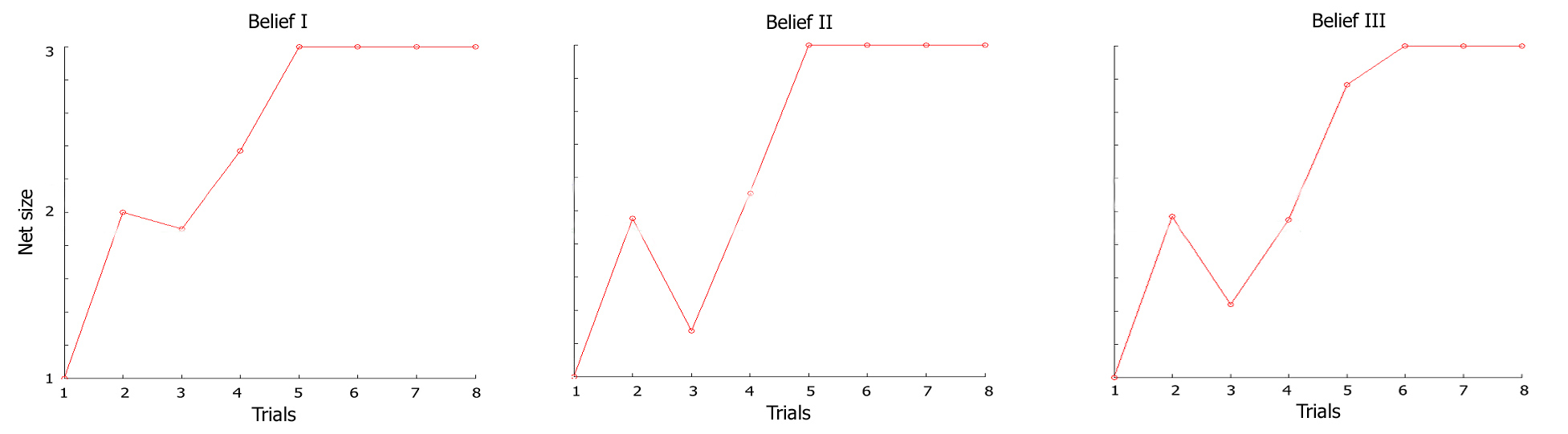


**Figure S1 |** The optimal behavioral strategy to maximize payoff in the non-social condition. Belief 1: assuming equal prior beliefs for the three possible migration rates. Belief 2: assuming beliefs corresponding to the actual migration rates. Belief 3: assuming equal prior beliefs for the three possible migration rates for the first round of all games and updating of these beliefs for the following rounds.


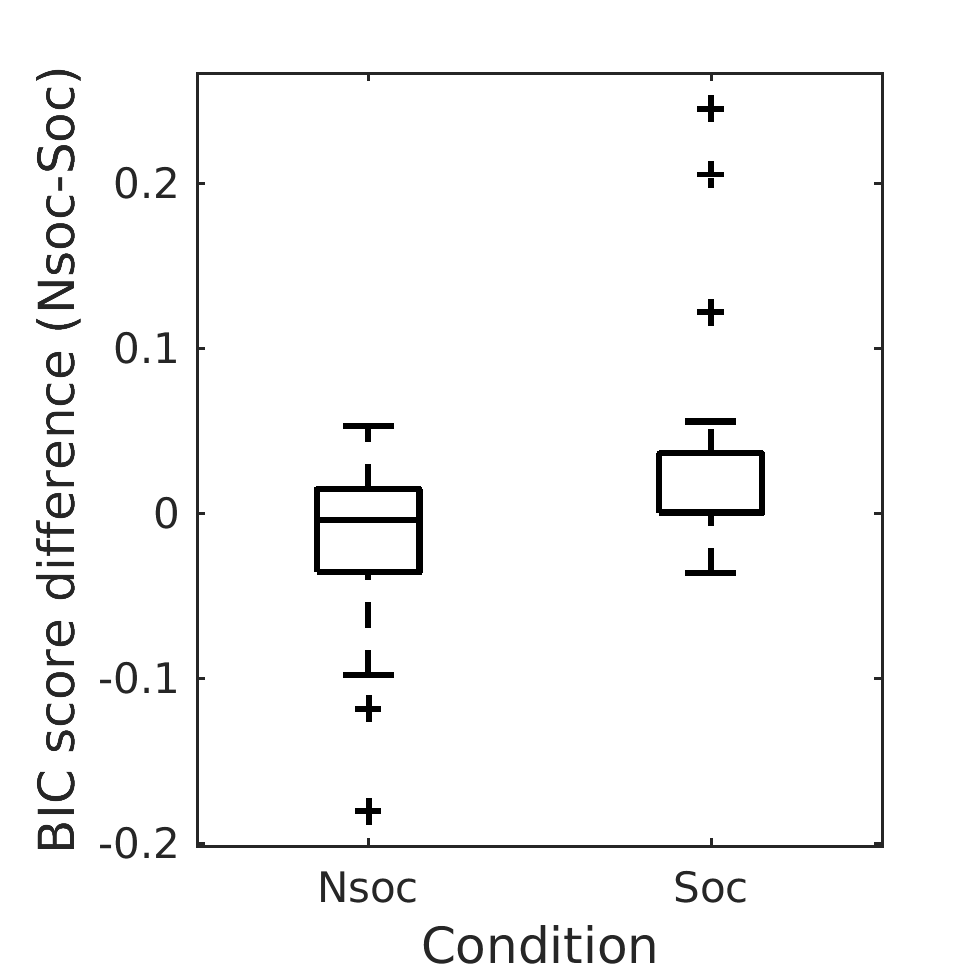


**Figure S2 |** BIC score differences between the social learning model and the non-social learning model grouped by social (Soc) and non-social (Nsoc) conditions, averaged across participants.


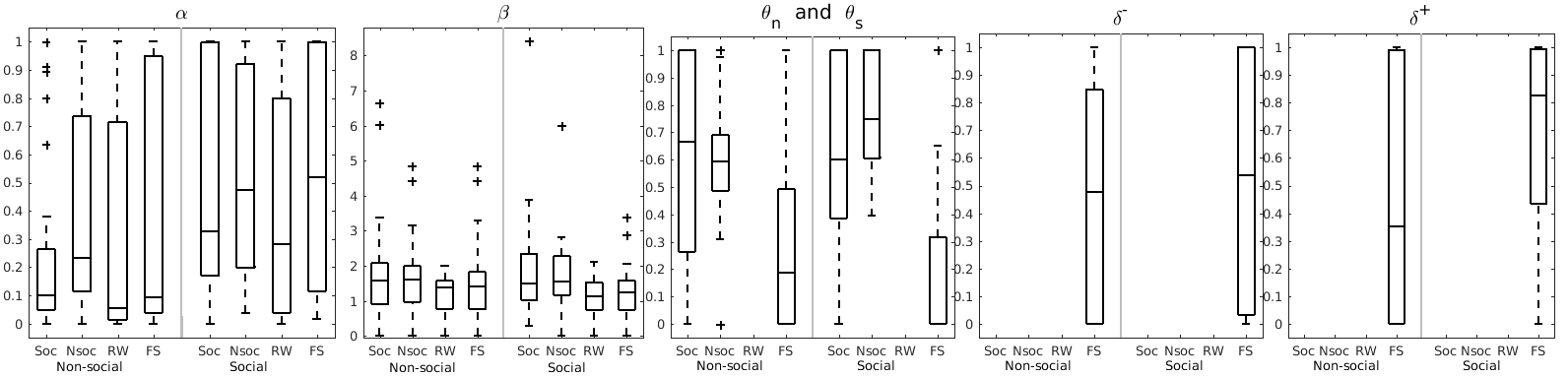


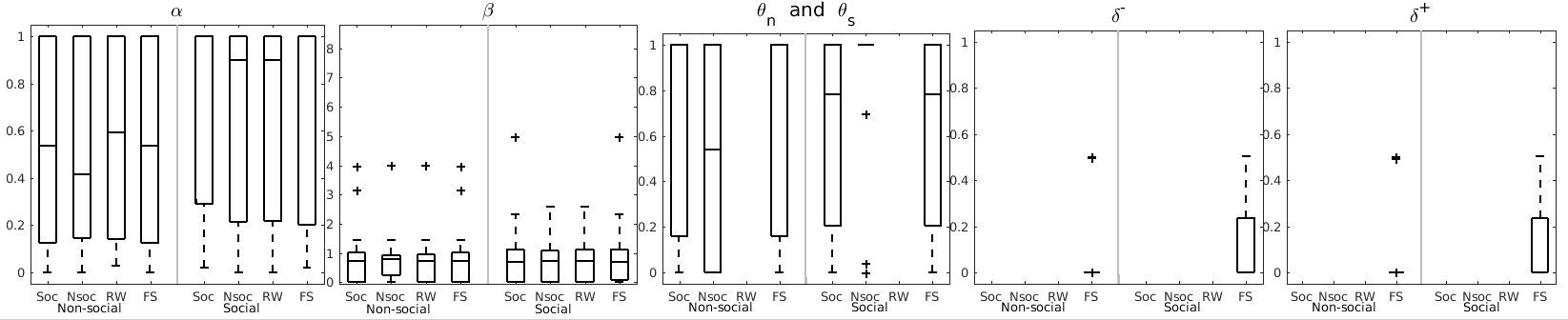


**Figure S3 |** Model parameter estimates. Top: estimates for behavioral data, across participants (N=50). Each column in a plot represents the learning algorithm (Soc: social; Nsoc: non-social; RW: Rescorla-Wagner; FS: Fehr-Schmidt) whose parameter was fitted. Bottom: estimates for simulated data, averaged across 50 simulated runs, for the 4 learning algorithms Soc, Nsoc, RW, FS. Learning rate (ɑ), inverse temperature (β), social comparison weight (θ_s_), sustainability weight (θ_n_), and advantageous (δ^+^) and disadvantageous inequality (δ^-^) coefficients (δ^+^ and δ^-^ pertain only to the Fehr-Schmidt inequity aversion model). Abscissa upper row labels indicate model type, and lower row labels indicate the experimental condition.


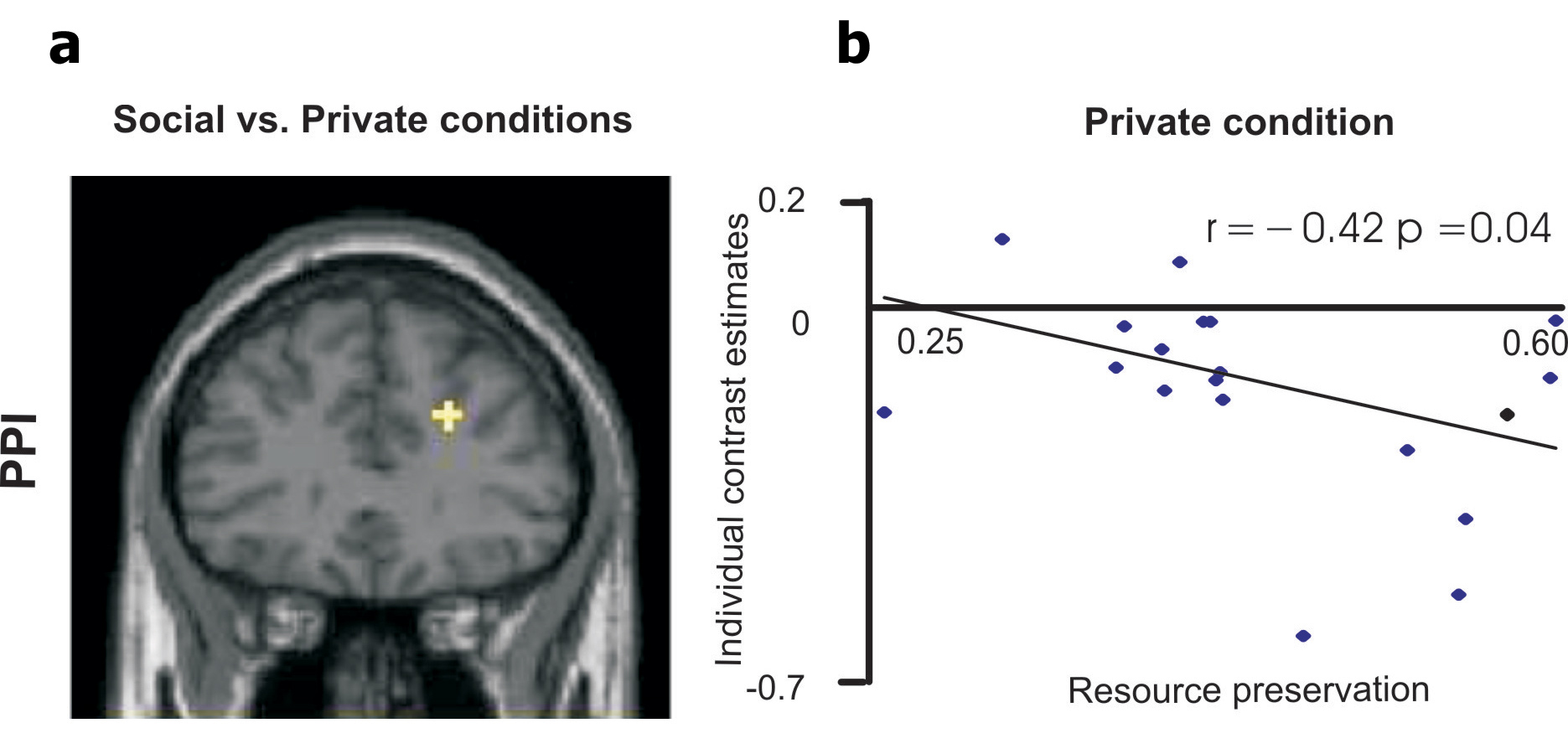


**Figure S4 |** Stronger functional connectivity between the right ventral striatum and the anterior dorsolateral prefrontal cortex (DLPFC) in the social condition. The PPI analysis was performed for the sharp depletion of the resource (sharp depletion < moderate depletion) and included all participants in both conditions. In the non-social condition, anterior DLPFC–ventral striatum connectivity was reduced as a result of a trend toward negative connectivity (A) Z-maps for PPI. (B) A trend toward negative connectivity of the right ventral striatum and the anterior DLPFC was observed in the non-social condition. The effect negatively correlated with resource preservation behavior. Map thresholded at P < .001, uncorrected.

**Table S1 |** ANOVA marginal tests for effect of social/non-social model type (ModelType) and group treatment (Condition) on BIC score.

| Term | F | DF1 | DF2 | p |
| --- | --- | --- | --- | --- |
| (Intercept) | 3291.4 | 1 | 110.7 | **3.25e-84** |
| ModelType | 3.576 | 4 | 192 | **7.73e-3** |
| Condition | .0018 | 1 | 110.7 | .966 |
| ModelType x Condition | 5.864 | 4 | 192 | **1.79e-4** |

**Table S2 |** Significant activation clusters to sharp resource depletion in both experimental conditions.

| Brain Region | x | y | z | No. of Voxels | Z |
| --- | --- | --- | --- | --- | --- |
| *moderate depletion of the resource < sharp depletion of the resource* | | | | | |
| Superior temporal gyrus, BA42 | 66 | -31 | 22 | 46 | 4.45 |
| Postcentral gyrus, BA43 | 51 | -13 | 19 | 43 | 3.83 |
|  |  |  |  |  |  |
| *moderate depletion of the resource > sharp depletion of the resource* | | | | | |
| Middle frontal gyrus, BA46 | -48 | 23 | 31 | 196 | 5.22 |
| Cerebellum | 42 | -67 | -41 | 356 | 4.74 |
| Cerebellum | -36 | -73 | -47 | 175 | 4.65 |
| Middle frontal gyrus, BA9 | 48 | 29 | 31 | 522 | 4.60 |
| Ventral striatum | -12 | 2 | -8 | 55 | 4.57 |
| Superior parietal lobule, BA7 | -33 | -52 | 49 | 277 | 4.41 |
| Ventral striatum | 9 | 5 | -5 | 128 | 4.39 |
| Inferior parietal lobule, BA40 | 42 | -43 | 46 | 258 | 4.27 |
| Superior frontal gyrus, BA10 | -30 | 59 | 10 | 154 | 4.10 |
| Cuneus, BA17 | 15 | -88 | 4 | 117 | 4.08 |
| Middle frontal gyrus, BA6 | 30 | 17 | 58 | 94 | 4.04 |
| Middle frontal gyrus, BA6 | -24 | 17 | 61 | 37 | 3.84 |
| Middle occipital gyrus, BA18 | -24 | -85 | -5 | 26 | 3.56 |
|  |  |  |  |  |  |

Local maxima within these clusters are reported together with the number of voxels (No. of Voxels); BA, Brodmann area; x, y, z are MNI coordinates of the local maximum.

**References**

- Fischbacher U (2007) z-Tree: Zurich toolbox for ready-made economic experiments.(Translated from English) Experimental Economics 10(2):171-178 (in English).
- Friston, K.J., Buechel, C., Fink, G.R., Morris, J., Rolls, E., & Dolan, R.J. (1997). Psychophysiological and modulatory interactions in neuroimaging. *NeuroImage, 6*(3), 218-229.
